# Systematic Analysis of *Hsf* Family Genes in the *Brassica napus* Genome Reveals Novel Responses to Heat, Drought and High CO_2_ Stresses

**DOI:** 10.3389/fpls.2017.01174

**Published:** 2017-07-06

**Authors:** Xiaoyi Zhu, Chunqian Huang, Liang Zhang, Hongfang Liu, Jinhui Yu, Zhiyong Hu, Wei Hua

**Affiliations:** Key Laboratory of Biology and Genetic Improvement of Oil Crops, Ministry of Agriculture, Oil Crops Research Institute of the Chinese Academy of Agricultural SciencesWuhan, China

**Keywords:** *Hsf* gene family, abiotic stress, high CO_2_, gene expression, *Brassica napus*

## Abstract

Drought and heat stress are major causes of lost plant crop yield. In the future, high levels of CO_2_, in combination of other abiotic stress factors, will become a novel source of stress. Little is known of the mechanisms involved in the acclimation responses of plants to this combination of abiotic stress factors, though it has been demonstrated that heat shock transcription factors (Hsfs) are involved in plant response to various abiotic stresses. In this study, we performed a genome-wide identification and a systematic analysis of genes in the *Hsf* gene family in *Brassica napus*. A total of 64 genes encoding Hsf proteins were identified and classified into 3 major classes: A, B and C. We found that, unlike in other eudicots, the A9 subclass is absent in rapeseed. Further gene structure analysis revealed a loss of the only intron in the DBD domain for *BnaHsf63* and -*64* within class C, which is evolutionarily conserved in all *Hsf* genes. Transcription profile results demonstrated that most *BnaHsf* family genes are upregulated by both drought and heat conditions, while some are responded to a high CO_2_ treatment. According to the combined RNA-seq and qRT-PCR analysis, the A1E/A4A/A7 subclasses were upregulated by both drought and heat treatments. Members in class C seemed to be predominantly induced only by drought. Among *BnaHsf* genes, the A2/A3/B2 subclasses were regulated by all three abiotic stresses. Members in A2/B2 subclasses were upregulated by drought and heat treatments, but were downregulated under high CO_2_ conditions. While the A3 subclass was upregulated by all the three abiotic stresses. Various stress-related *cis*-acting elements, enriched in promoter regions, were correlated with the transcriptional response of *BnaHsfs* to these abiotic stresses. Further study of these novel groups of multifunctional *BnaHsf* genes will improve our understanding of plant acclimation response to abiotic stresses, and may be useful for improving the abiotic stress resistance of crop varieties.

## Introduction

Environmental stresses, such as drought and heat, cause substantial loss to plant growth and production (Hu and Xiong, 2014; Fang and Xiong, 2015; Pereira, 2016; Zhu, 2016). Increasing CO_2_ levels result in lower concentrations of zinc, iron and protein for most C3 crops, and threaten human food sources; these results suggest that high CO_2_ concentrations (hereafter abbreviated to high CO_2_) may be a novel stress in the future (Myers et al., 2014). However, the basic molecular mechanisms driving plant responses to high CO_2_ remain elusive (Becklin et al., 2017). To cope with abiotic stresses, plants have evolved diverse adaptive strategies and signaling mechanisms. Transcription factors play crucial regulatory roles in the signal transduction process under these stresses (Hu and Xiong, 2014; Yoshida et al., 2014; Song et al., 2016). Among the transcription factors, heat shock transcription factors (Hsfs) serve as the terminal components in the signal transduction chain mediating the activation of genes responsive to heat and other stresses by recognizing palindromic binding motifs conserved in promoters of heat stress inducible genes called heat stress elements (HSEs: 5′-AGAAnnTTCT-3′) (von Koskull-Döring et al., 2007; Scharf et al., 2012; Guo et al., 2016).

As many other transcription factors, Hsf proteins possess a conserved modular structure (von Koskull-Döring et al., 2007; Scharf et al., 2012). The highly-structured DNA-binding domain (DBD) is located close to the N-terminal of all Hsfs and is responsible for selective interaction with HSE. An oligomerization domain (OD or HR-A/B region) is connected to the DBD by a flexible linker of variable length (15–80 amino acid residues) with a bipartite heptad pattern of hydrophobic amino acid residues, leading to the formation of a coiled-coil domain for protein interaction of Hsfs. Based on the length of the flexible linker and the number of amino acid residues inserted into the HR-A/B region, plant Hsfs are grouped into three main classes (HsfA, -B, and -C) (von Koskull-Döring et al., 2007; Scharf et al., 2012; Guo et al., 2016). The nuclear localization signal (NLS) and the nuclear export signal (NES) control the intracellular distribution of Hsfs between nucleus and cytoplasm. AHA motifs are usually present in the *HsfA* subfamily and confer transcriptional activator function. However, HsfB members, except HsfB5, are characterized by a repressor domain (RD) consisting of tetrapeptide LFGV in the C-terminal region.

Heat shock transcription factor genes were first cloned in yeast in 1988 (Wiederrecht et al., 1988). Unlike the small Hsf family in yeast and animals, plants hold complex and large *Hsf* gene families. 21, 25, 38, 40, 25, 32 Hsf genes have been found in *Arabidopsis*, rice, soybean, cotton, pepper and poplar, respectively (Nover et al., 2001; Chauhan et al., 2011; Li et al., 2014; Wang et al., 2014; Guo et al., 2015; Zhang et al., 2016). Among monocots and eudicots, the largest number of *Hsf* genes were found in wheat (56 members) (Xue et al., 2014).

Plant *Hsfs* do not only play a role in heat stress response, but also function both in response to other stressers and during organ development. In addition, structural characteristics and diverse expression patterns of *Hsf* family genes have revealed functional diversification (von Koskull-Döring et al., 2007). Our understanding of plant *Hsf* gene function comes predominantly from studies of tomato and *Arabidopsis thaliana*. In tomato, constitutively expressed *HsfA1a* functions as a master regulator for induced thermotolerance, and cannot be replaced by any other *Hsfs* (Mishra et al., 2002). However, in *Arabidopsis*, due to functional redundancy no comparable master regulator role could be identified for any of its four HsfA1 genes (Liu et al., 2011; Nishizawa-Yokoi et al., 2011; Guo et al., 2016). HsfA2 is structurally and functionally similar to HsfA1, but is only strongly accumulated in stressed plants. Its interaction with HsfA1 and B1 forms a functional complex, responsible for regulating core aspects of heat stress response and recovery (Nishizawa et al., 2006; Charng et al., 2007; Scharf et al., 2012; Guo et al., 2016). Additionally, HsfA2 is also involved as a key regulator in other environmental stresses such as osmotic and oxidative stress (Ogawa et al., 2007; Wang et al., 2017). Previous investigations showed that heat- and drought-induced expression of *HsfA3* depends on transcription factor DREB2A in *Arabidopsis*, this indicates that *HsfA3* plays a role in drought stress signaling (Sakuma et al., 2006; Schramm et al., 2008). Ectopic expression of *HsfA3* has also been shown to enhance thermotolerance in *Arabidopsis* (Li et al., 2013). Group A4 *Hsfs* are involved in controlling reactive oxygen species homeostasis of plants, and group A5 *Hsfs* act as specific repressors of *HsfA4* (Yamanouchi et al., 2002; Baniwal et al., 2007). Orthologous *HsfA4a* was reported to confer cadmium tolerance in wheat and rice (Shim et al., 2009). *HsfA9* participates in embryo development and seed maturation in *Arabidopsis* and sunflower (Almoguera et al., 2002; Kotak et al., 2007); in addition, overexpression of seed-specific *HaHSFA9* in tobacco confers tolerance to severe dehydration at vegetative stage (Prieto-Dapena et al., 2008).

Unlike class A *Hsfs*, class B *Hsfs* lack an AHA motif and show no independent function as transcriptional activators (von Koskull-Döring et al., 2007). However, in tomato, heat-induced *HsfB1* acts as a coactivator with *HsfA1a* (Bharti et al., 2004). Moreover, Arabidopsis *HsfB1* is inactive as a coactivator, although *AtHsfB1* can act as a repressor of heat stress-inducible Hsfs (Czarnecka-Verner et al., 2000, 2004). To date, limited information is available for class C *Hsfs*. The results from tomato and *Arabidopsis* revealed striking species-specific deviations in the functional diversification of *Hsf* family members (von Koskull-Döring et al., 2007; Scharf et al., 2012; Guo et al., 2016). Overall, comprehensive characterization is still needed to investigate the functions of *Hsfs* in plant abiotic stress responses, and in particular in response to high CO_2_ conditions.

*Brassica napus* (rapeseed) is one of the most important oil crops in the world. Given the recent publication of the *B. napus* genome (Chalhoub et al., 2014), rapeseed is becoming an important crop model system (Zhu et al., 2016). While rapeseed plants are sensitive to water deficit and high temperature during all stages of growth and development, the typical stress-related Hsf transcription factors have not yet been characterized in *B. napus*. To investigate the potential roles of rapeseed *Hsfs* in abiotic stress responses, the present study identifies 64 genes encoding BnaHsf proteins in the *B. napus* genome and analyzes their phylogenetic relationships, gene and domain structures, putative *cis*-acting elements, and expression patterns across different tissues and under heat, drought and high CO_2_ stresses. The results of this study will help to provide a foundation for further functional studies of *BnaHsf* genes, and improve our understanding of the functional diversification of plant *Hsf* genes under various environmental stresses.

## Materials and Methods

### Identification of *Hsf* Genes in *Brassica napus*

The gene sequence of *B. napus* were downloaded from the genome database^1^, to gather the probable candidate *B. napus* Hsf amino acid sequences, the Hsf-type DBD domain (Pfam: PF00447) was submitted as a query in a protein BLAST search of the *B. napus* genome database. *Hsf* gene sequences from *Arabidopsis* (Nover et al., 2001) were retrieved from the TAIR database (Lamesch et al., 2012) and used as queries to perform repetitive BLAST searches against the Phytozome database v9.1 (Goodstein et al., 2012). BLAST searches were also performed against the NCBI nucleic-acid sequence data repositories. All protein sequences derived from the BLAST searches were examined using domain-analysis programs. Molecular weight, iso-electric point, functional domains, and amino acid signal peptides of BnaHsfs were calculated using the ExPASy online servers^2^.

### Multiple Sequence Alignments and Phylogenetic Analysis

Multiple sequence alignment of Hsf proteins from *B. napus* were performed using the program ClustalX 1.83 (Thompson et al., 1997). The phylogenetic tree was constructed using the neighbor-joining (NJ) method by MEGA 6 program (Tamura et al., 2013), the bootstrap value was set at 1000 replications to assess tree reliability.

### Domain and Gene Structure Analysis

The MEME program^3^ (Bailey et al., 2009) was used for identification of conserved motifs, with the following parameters: number of repetitions: any; maximum number of motifs:15; and the optimum motif widths: 6–200 amino acid residues. Exton/intron organization of the *Hsf* genes in *B. napus* was illustrated using Gene Structure Display Server program (GSDS^4^) (Hu et al., 2015) by alignment of the cDNAs with their corresponding genomic DNA sequences.

### Regulatory *Cis*-Element Analysis

Prediction of putative *cis*-elements was performed using Signal Scan Search against the plant *cis*-acting regulatory DNA elements (PLACE) database (Higo et al., 1999). A 2-kb sequence upstream of ATG initiation codon of *BnaHsf* genes was selected as the promoter region for this analysis.

### Plant Materials and Growth Conditions

Rapeseed seeds were germinated on a filter paper, and then transplanted to soil pots growing in the greenhouse at Oil crops research institute (Wuhan, China) with conditions of a temperature of 22°C, LED sodium lamp and a humidity of about 50–70% for 6 weeks. The plants were then transferred to growth chamber programmed under specific environmental conditions for 2–3 days before stress treatment. The conditions in growth chamber were set as follows: temperature of 25°C and humidity of 50% in 16 h light; temperature of 22°C and humidity of 60% in 8 h dark.

### Stress Treatments

The high CO_2_ stress was performed in a growth chamber (AR-41L2, United States) in which CO_2_ gas can be accurately and stably controlled in the range of 100–3000 ppm. The conditions of growth chamber were set as follows: CO_2_ concentration of 1000 ppm, light intensity of 100 umol/m^2^/s, temperature of 25°C and 60% relative humidity. Leaf samples were collected at 1, 3, and 6 h during the treatment.

The heat and drought stress were performed in a common growth chamber. For heat stress, the chamber was set with temperature of 40°C and humidity of 60%. Leave samples under heat were collected at 1, 3, and 6 h during treatment. For drought stress, the chamber was set as follows: temperature of 25°C and humidity of 40% in 16 h light; temperature of 22°C and humidity of 50% in 8 h dark; withholding water for 7 days, leaf samples were collected at 1, 2, and 3 days during drought treatment.

All the collected leaf samples were immediately frozen in liquid nitrogen, and stored at -70°C for further analysis.

### RNA Isolation and Quantitative Real-Time PCR (qRT-PCR) Analysis

The RNA was isolated from leaf tissues using an RNA extraction kit (Takara, Dalian), according to the manufacturer’s instructions. The first-strand cDNA was synthesized by the Prime Script RT reagent Kit (Takara, Dalian). Real-time quantitative PCR was performed using 2 μl of cDNA in a 20 μl reaction volume with SYBR Premix Ex Taq (Takara) on a 7500-Fast real-time PCR System (Applied Biosystems). Gene-specific primers were designed (Supplementary Table 1). The rapeseed *TMA7* gene (BnaC05g11560D) was used as an internal control to normalize the expression level of the target gene, which has highly stable expression level in different tissues and under various growth conditions. Each treatment was repeated three times independently. The thermal cycler was set as follows: an initial incubation at 50°C for 2 min and 95°C for 5 min, followed by 40 cycle at 95°C for 30 s, 55°C for 30 s and 72°C for 30 s. The relative quantification of *BnaHsfs* transcription levels was determined by the methods described previously (Livak and Schmittgen, 2001).

## Results

### Genome-Wide Identification of *Hsf* Genes in *Brassica napus*

To identify *Hsf* genes in *B. napus*, candidate genes were selected by using the conserved Hsf domain (PF00447) from the Pfam database to query the *B. napus* genome. Meanwhile, the amino acid sequences of 21 AtHsfs from *Arabidopsis* were used to protein BLAST the *B. napus* genome. A total of 64 *BnaHsfs* (*BnaHsf01*-*BnaHsf64*) were identified as members of the *Hsf* gene family in *B. napus*, through simultaneous consideration of the conservation of the DBD domain, the coiled-coil structure from the SMART database, and the CD-search of the NCBI database (**Table 1**).

**Table 1:** Summary information of the *BnaHsf* family genes in *Brassica napus.*

Gene name	Subfamily	Gene ID	Size (aa)	pI	MW (kDa)	Chromosome location
*BnaHsf01*	A1A	BnaA01g08640D	430	5.06	47.82	chrA01:4144293..4145692
*BnaHsf02*		BnaCnng36910D	437	5.01	48.37	chrCnn_random:35221874..35223412
*BnaHsf03*	A1B	BnaA02g03270D	442	4.84	49.00	chrA02:1454367..1456043
*BnaHsf04*		BnaC02g06880D	439	4.74	48.81	chrC02:3678716..3680294
*BnaHsf05*		BnaA10g17670D	487	4.92	53.69	chrA10:12997867..13000101
*BnaHsf06*		BnaC09g41040D	441	4.86	48.71	chrC09:43210122..43212302
*BnaHsf07*	A1D	BnaA08g06910D	475	4.61	53.01	chrA08:6951258..6963650
*BnaHsf08*		BnaA09g24420D	487	4.65	54.41	chrA09:17142341..17145257
*BnaHsf09*		BnaC05g24440D	492	4.67	55.01	chrC05:18915447..18918268
*BnaHsf10*	A1E	BnaA03g28190D	455	5.54	50.33	chrA03:13771476..13773786
*BnaHsf11*		BnaC03g33280D	448	5.97	49.45	chrC03:20319811..20322126
*BnaHsf12*		BnaA05g32280D	306	8.58	34.64	chrA05:22119903..22121886
*BnaHsf13*		BnaCnng06170D	436	5.14	48.45	chrCnn_random:5550634..5553157
*BnaHsf14*		BnaAnng15230D	454	5.22	50.35	chrAnn_random:16329216..16331988
*BnaHsf15*	A2	BnaA03g22890D	373	5.35	41.74	chrA03:10896133..10898064
*BnaHsf16*		BnaC03g26940D	349	4.99	39.23	chrC03:15484198..15486234
*BnaHsf17*	A3	BnaA10g26390D	363	5.03	41.14	chrA10:16827004..16829101
*BnaHsf18*		BnaCnng02620D	401	5.00	45.00	chrCnn_random:2245207..2247371
*BnaHsf19*	A4A	BnaA01g09690D	517	5.81	58.24	chrA01:4758848..4761452
*BnaHsf20*		BnaC01g11370D	384	5.13	44.19	chrC01:7036485..7037836
*BnaHsf21*		BnaA08g08940D	366	5.22	41.89	chrA08:8674028..8675413
*BnaHsf22*		BnaC03g62890D	362	5.13	41.71	chrC03:52189057..52190616
*BnaHsf23*		BnaC07g35520D	393	5.24	45.01	chrC07:37919226..37920772
*BnaHsf24*		BnaAnng31620D	390	5.35	44.93	chrAnn_random:36194905..36196930
*BnaHsf25*	A4C	BnaA09g17760D	307	5.73	35.21	chrA09:10918958..10920504
*BnaHsf26*		BnaC09g18620D	340	5.64	39.03	chrC09:15379926..15381736
*BnaHsf27*	A5	BnaA04g06390D	477	5.93	53.03	chrA04:4997155..4999196
*BnaHsf28*		BnaC04g29180D	475	5.60	52.69	chrC04:30523561..30525671
*BnaHsf29*	A6A	BnaA02g22350D	262	5.62	31.06	chrA02:14861621..14862576
*BnaHsf30*	A6B	BnaCnng14280D	383	5.13	44.11	chrCnn_random:13105130..13107008
*BnaHsf31*		BnaCnng14290D	371	5.60	43.11	chrCnn_random:13113098..13114967
*BnaHsf32*		BnaA01g24460D	379	5.18	43.89	chrA01:16815884..16817720
*BnaHsf33*		BnaA05g16880D	389	4.89	45.03	chrA05:11618772..11620931
*BnaHsf34*		BnaC05g29680D	388	4.89	44.93	chrC05:28509649..28511854
*BnaHsf35*	A7A	BnaC04g28450D	268	5.89	31.15	chrC04:29839491..29841118
*BnaHsf36*		BnaA03g41540D	287	6.58	33.55	chrA03:20863324..20868014
*BnaHsf37*		BnaA03g41550D	269	5.53	31.37	chrA03:20868066..20869387
*BnaHsf38*		BnaC07g32600D	265	5.63	30.94	chrC07:36175409..36176731
*BnaHsf39*	A7B	BnaA09g40360D	285	5.79	33.28	chrA09:28417721..28419329
*BnaHsf40*		BnaC08g32790D	285	5.97	33.18	chrC08:31602538..31604220
*BnaHsf41*	A8	BnaA07g26740D	384	4.82	43.71	chrA07:19580905..19582658
*BnaHsf42*		BnaC06g29140D	380	4.90	43.03	chrC06:30205027..30206798
*BnaHsf43*		BnaC02g17710D	362	4.87	41.05	chrC02:13307394..13309102
*BnaHsf44*	B1	BnaA03g53750D	271	5.85	30.04	chrA03:28301205..28302239
*BnaHsf45*		BnaCnng56320D	272	5.55	30.20	chrCnn_random:56246881..56248020
*BnaHsf46*		BnaC01g01790D	284	6.05	31.18	chrC01:921667..922872
*BnaHsf47*		BnaAnng36200D	286	6.05	31.32	chrAnn_random:41072330..41073455
*BnaHsf48*	B2A	BnaA06g21470D	293	6.56	32.87	chrA06:14903025..14904155
*BnaHsf49*		BnaC03g52080D	300	6.56	33.59	chrC03:36862475..36863579
*BnaHsf50*		BnaCnng54110D	252	6.99	28.35	chrCnn_random:53865699..53866743
*BnaHsf51*	B2B	BnaA03g24840D	320	4.86	34.67	chrA03:11996865..11998262
*BnaHsf52*		BnaC03g73070D	310	4.99	33.85	chrC03_random:1225902..1227717
*BnaHsf53*		BnaA09g21510D	361	4.66	38.21	chrA09:14189356..14190930
*BnaHsf54*		BnaC09g52680D	361	4.66	38.21	chrC09_random:2042369..2043875
*BnaHsf55*	B3	BnaA03g19560D	239	5.17	27.92	chrA03:9269373..9270563
*BnaHsf56*		BnaC03g23450D	226	5.88	26.42	chrC03:13066673..13068936
*BnaHsf57*	B4	BnaA08g04110D	335	9.16	38.15	chrA08:3451960..3453065
*BnaHsf58*		BnaC08g04780D	335	9.16	38.33	chrC08:5463460..5464644
*BnaHsf59*		BnaA10g05440D	329	8.71	37.39	chrA10:3141594..3142758
*BnaHsf60*		BnaC06g00310D	330	7.85	37.67	chrC06:491206..492436
*BnaHsf61*	C	BnaA07g05580D	314	5.87	35.79	chrA07:5882469..5883727
*BnaHsf62*		BnaC07g07130D	314	5.87	35.91	chrC07:11406343..11407422
*BnaHsf63*		BnaA03g37460D	306	6.57	34.90	chrA03:18569380..18570300
*BnaHsf64*		BnaC03g43990D	315	6.06	35.71	chrC03:29056023..29056970

Fifty-four *BnaHsf* genes were distributed unevenly among the 19 chromosomes of *B. napus* from A01 to A10 and C01 to C09; however, 10 members (including *BnaHsf02*) were located on unanchored scaffolds that could not be mapped to a specific chromosome. Most *BnaHsf* genes were localized to chromosome A03 and C03 (8 and 7 *Hsf* genes), while there was only one *BnaHsf* on chromosome A04 and A06 (**Table 1**). The deduced proteins of the *BnaHsfs* ranged from 226 amino acids (aa; *BnaHsf56*) to 517 aa (*BnaHsf19*) in length, with predicted isoelectric points (pI) varying from 4.61 (*BnaHsf07*) to 9.16 (*BnaHsf57* and *BnaHsf60*) and molecular weight (MW) from 26.42 kDa (*BnaHsf56*) to 58.24 kDa (*BnaHsf19*) (**Table 1**).

### Phylogenetic Analysis and Multiple Sequence Alignment of BnaHsf Proteins

**FIGURE 1:**
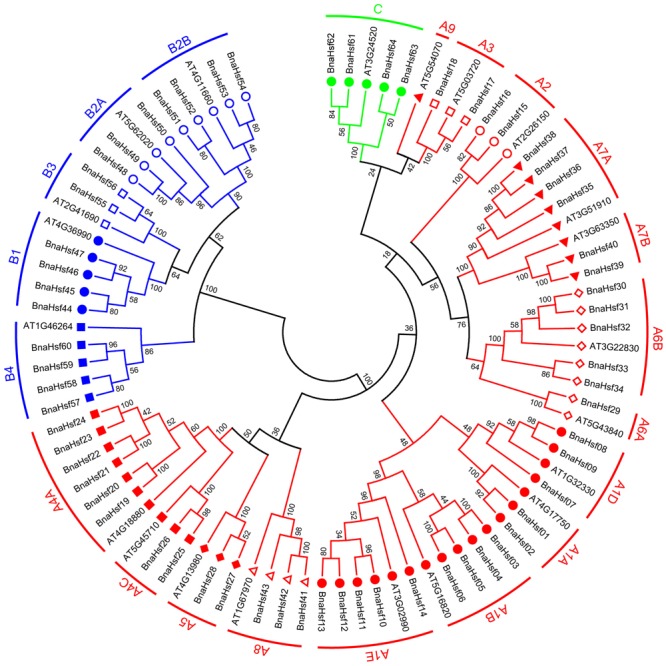
Unrooted phylogenetic tree of rapeseed and *Arabidopsis* Hsf family. Amino acid sequences of Hsf proteins were analyzed using the neighbor-joining method with genetic distance calculated by MEGA6.0. The numbers at the nodes represent percentage bootstrap values based on 1,000 replications. The length of the branches is proportional to the expected number of amino acid substitutions per site.

Among the best-studied 21 *Arabidopsis* Hsfs, 15 members belong to group A and 5 members belong to Group B, and only one Hsf has been classified as part of group C (Nover et al., 2001). To explore the classification and the evolutionary characteristics of the *BnaHsf* genes, an unrooted phylogenetic tree was generated using protein sequences of *BnaHsfs* (**Figure 1** and Supplementary Datasheet 1).

According to this phylogenetic analysis, the *BnaHsfs* genes were grouped into three classes, A (*BnaHsf01*-*BnaHsf43*), B (*BnaHsf44*-*BnaHsf60*), and C (*BnaHsf61*-*BnaHsf64*), as in *Arabidopsis*. Class A was the largest and consisted of 43 members from eight subclasses (A1–A8). In *Arabidopsis*, class A is further subdivided into 9 subclasses, A1-A9, with A9 (At5g54070) appearing as a single branch of the *AtHsfs* molecular phylogeny. However, no orthologous *HsfA9* genes were found in *B. napus*, indicating that *Hsf* genes in this subgroup were lost. Class B consisted of 17 members from four subclasses (B1–B4), and class C was the smallest, containing only 4 members (**Figure 1** and **Table 1**).

**FIGURE 2:**
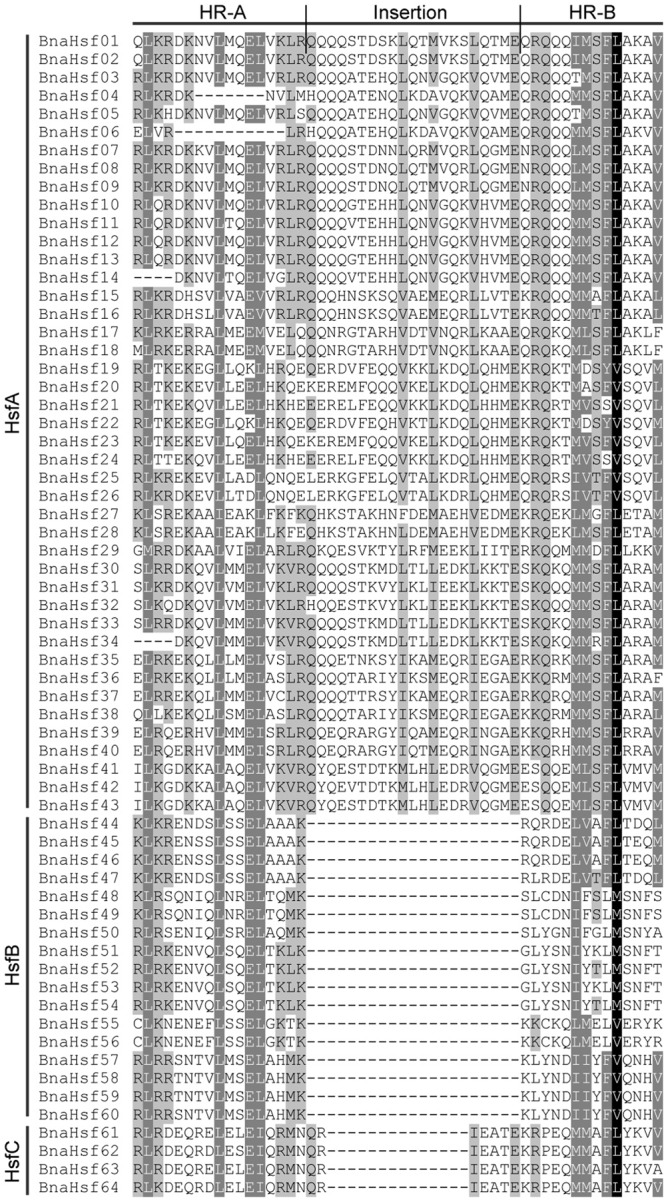
Multiple sequence alignment of the HR-A/B regions (OD domain) of rapeseed Hsf proteins.

Multiple sequence alignment analysis of BnaHsfs proteins showed that a typical *B. napus* Hsf protein contains 5 conserved domains, including of DBD, OD, NLS, NES, and AHA domains in order from N-terminal to C-terminal. Among these, the highly structured DBD and OD domains are the most conserved sections in each BnaHsf. The OD domain (HR-A/B region) served as an important basis for classification of *BnaHsfs*. *B. napus* class B Hsf proteins, like other plant Hsf proteins, are compact and lack an insertion between the HR-A and HR-B regions (**Figure 2**), while an insertion of 21 aa in length was found in *BnaHsfA* and an insertion of 7 aa in length was found in *BnaHsfC* between the HR-A and HR-B regions. Thus, class A members are less conserved than class B and C members.

### Structure and Motif Analysis of BnaHsf Proteins

**FIGURE 3:**
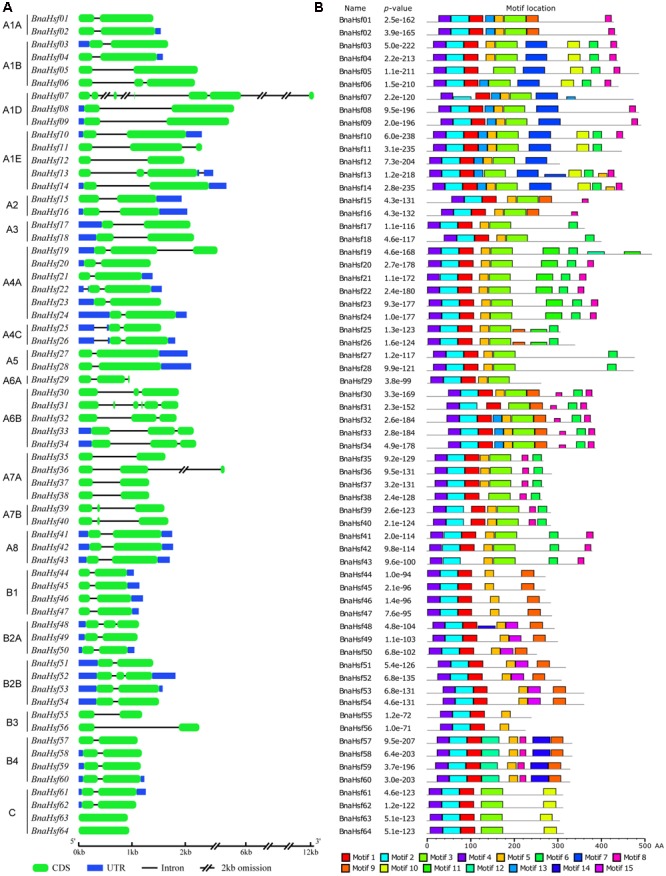
Gene organization of *BnaHsfs* **(A)** and motifs identified by MEME tools in rapeseed Hsf proteins **(B)**. Fifteen motifs (1–15) were identified and are here indicated by different colors. The combined *p*-value is shown.

To study the structural diversity of *BnaHsf* genes, the exon/intron organization of individual *BnaHsf* genes was analyzed by comparing cDNA sequences with the corresponding genomic DNA sequence. The detailed gene structures are shown in **Figure 3A**. The number of introns ranges from zero to five in *BnaHsf* genes. Five introns were found in *BnaHsf31*, while none were found in the *BnaHsf63*/*64* gene pair. Most *BnaHsfs* contained one or two introns (**Figure 3A**).

Conserved motif analysis was conducted by using MEME, and 15 motifs were detected in BnaHsf proteins (**Figure 3B** and Supplementary Figure 1). The DBD and OD domains, composed of motif 4, motif1 and motif 2, were the most conserved and were found in almost all 64 *BnaHsf* members, while motif 1 was absent in BnaHsf43. The NLS motif (motif 9) and NES motif (motif 15) were found in most class A and B members but not in class C BnaHsf proteins. Motif 3 is the insertion between the HR-A and HR-B regions that was found only in class A and class C members. The AHA motif (motif 6) was found in most class A members but not in classes B and C. In general, the structure of Hsf proteins was conserved throughout the *BnaHsfs* gene family.

### Tempo-Spatial Expression Profiles of *Hsf* Genes in *Brassica napus*

To examine spatial and temporal expression profiles of BnaHsfs across different tissues and organs, an expression pattern map was drawn based on RNA-seq data (Supplementary Table 2) from twelve rapeseed tissues (leaf, root, stem, sepal, silique, pericarp, bud, stamen, ovule, new pistil, mature pistil and wilting pistil).

**FIGURE 4:**
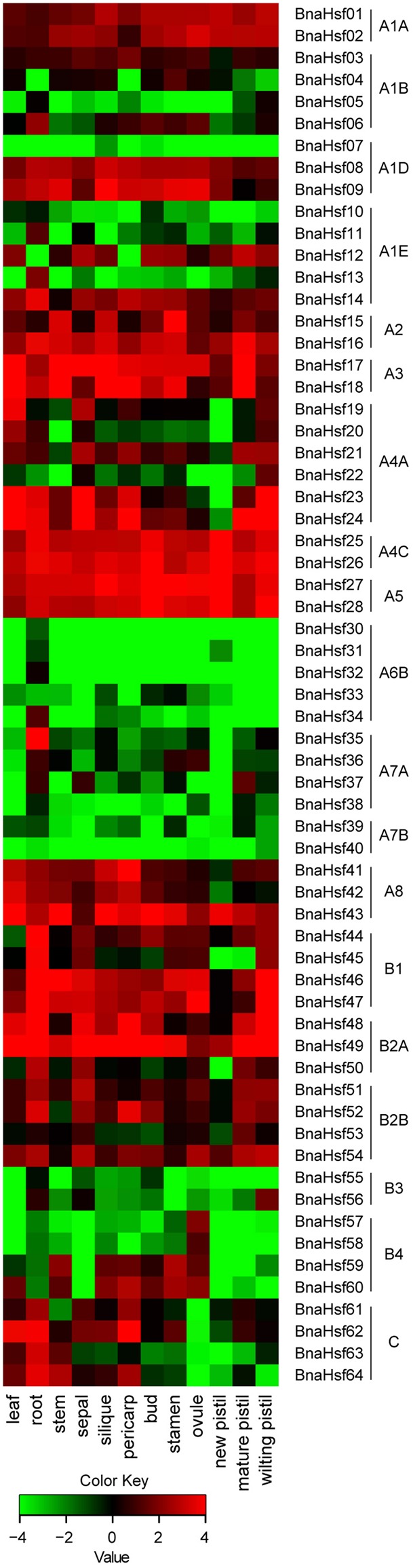
The tempo-spatial transcription profiles of *BnaHsf* family genes in various tissues at different developmental stages. The different colors correspond to log2 transformed value.

We found that *BnaHsf* genes were differently expressed among the subclasses in 14 tissues and at different developmental stages (**Figure 4**). *BnaHsfA6* subclass (*BnaHsf29*-*BnaHsf34*) exhibited root specific expression but was hardly detected in other tissues. Subclass A7 (*BnaHsf35*-*BnaHsf40*) was also root specific but *BnaHsf35* and *BnaHsf37* were also detected in reproductive organs such as petal, stamen and pistil. The *BnaHsf* A2, A3, A4C and A5 subclasses were the most abundant *BnaHsf* genes and were constitutively expressed among all tissues, as were *BnaHsf23* and -*24* in subclass A4A, *BnaHsf46* and -*47* in subclass B1, and *BnaHsf48* and -*49* in subclass B2A. Subclass B3 (*BnaHsf55*-*56*) showed a higher transcription level in root, sepal and bud tissues. *BnaHsf55* and -*56* in subclass B4 were specifically expressed in ovule tissues. In contrast, class BnaHsfC was inactive in ovule tissues.

### Expression Patterns of *BnaHsf* Genes under Abiotic Stresses

**FIGURE 5:**
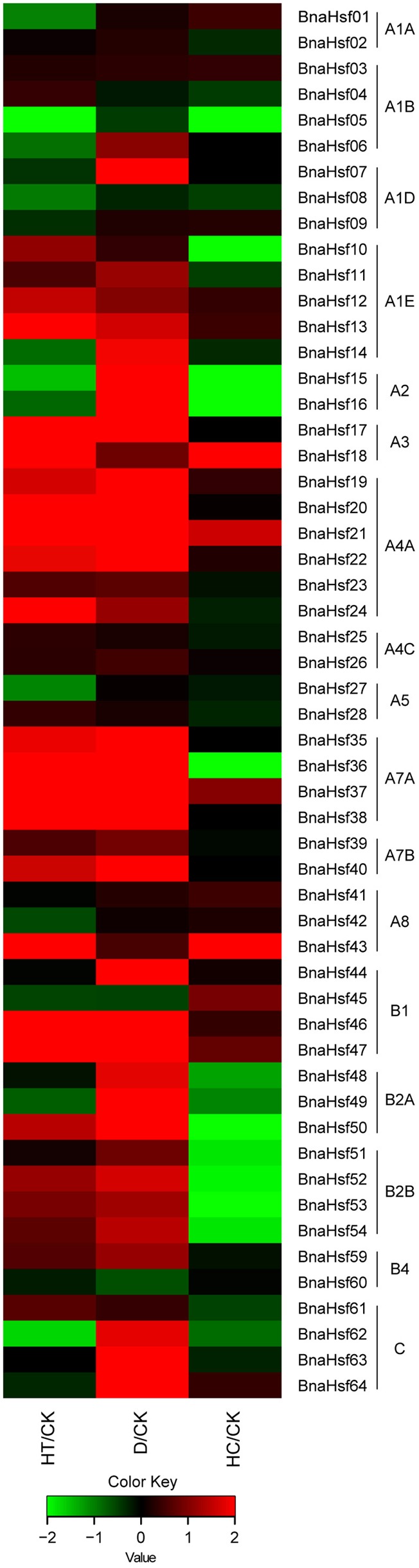
Expression profiles of *BnaHsf* genes under drought, heat and high CO_2_ conditions. HT/CK, expression levels under heat vs. control; D/CK, expression levels under drought vs. control; HC/CK, expression levels under High CO_2_ vs. control. The different colors correspond to log2 transformed value.

To determine the potential role *BnaHsfs* play in plant responses to different environmental stresses, the expression levels of *BnaHsf* genes under high temperature, drought and high CO_2_ stresses were analyzed using RNA-seq data (Supplementary Table 3). The mRNA for these transcriptomic analyses were extracted from the leaves of rapeseed plants both in normal growth conditions and after 3 h of heat treatment, 3 days of drought treatment and 3 h of high CO_2_ treatment. The results showed that *BnaHsf* genes were very sensitive to heat and drought stress (**Figure 5**). Members of subclasses A2, A4C, A5, and class B (except for the non-expressed subclasses B3 and B4) showed relatively higher basic transcription levels in leaf tissue under normal growth conditions. Among the subclasses with high basic expression levels, *BnaHsf15* and -*16*, in subclass A2, were dramatically upregulated (>25-fold), becoming the predominant transcripts after 3 days of exposure to drought stress, but were suppressed after 3 h of heat treatment. B1 members, except for *BnaHsf45*, were strongly induced by drought stress, and *BnaHsf46* and -*47* were strongly induced by heat. All B2 members were significantly upregulated under drought conditions, but were only slightly induced by 3 h of heat treatment. As observed in the subclasses with low basic expression, a moderate induction was seen in the A1E subclass after exposure to drought and heat. Members of subclasses A3 and A4A were strongly induced by both drought and heat stress. Strikingly, the highest induction (>350-fold for *BnaHsf36*∼*38*) by drought was observed in A7A subclass, although expression of members of this subclass was hardly detectable under normal conditions. Heat treatment also resulted in a marked induction in A7A members. Members in class C were only upregulated by drought treatment. In the case of individual member genes, *BnaHsf07* (in A1D) and *BnaHsf40* (in A7B) were induced by drought stress, and *BnaHsf43* in subclass A8 was strongly induced by heat stress.

Unlike drought and heat stress, high CO_2_ treatment did not cause a significant effect on the transcription level of most of the *BnaHsf* family genes (**Figure 5**). However, three member genes, *BnaHsf18* (in A3), *BnaHsf21* (in A4A) and *BnaHsf43* (in A8), were clearly induced (3 to 9-fold) by exposure to high CO_2_ conditions. However, the expression of members of subclass A2 members (*BnaHsf15*/*16*) and of subclass B2 was largely suppressed by high CO_2_ treatment.

### qRT-PCR Expression Analysis of Selected *BnaHsf* Genes under Abiotic Stresses

Twelve *BnaHsf* genes from three main classes were selected for examination of their function under three stress conditions using quantitative Real-Time PCR (qRT-PCR). These genes included *BnaHsf04* from subclass A1, *BnaHsf15* and -*16* from subclass A2, *BnaHsf17* and -*18* from subclass A3, *BnaHsf20* and *BnaHsf23* from subclass A4, *BnaHsf42* from subclass A8, *BnaHsf45* from subclass B1, *BnaHsf46* from subclass B2 and *BnaHsf61* from class C. qRT-PCR was carried out using rapeseed plants exposed to heat (0, 1, 3, and 6 h), drought (0, 1, 2, and 3 days), and high CO_2_ (0, 1, 3, and 6 h).

**FIGURE 6:**
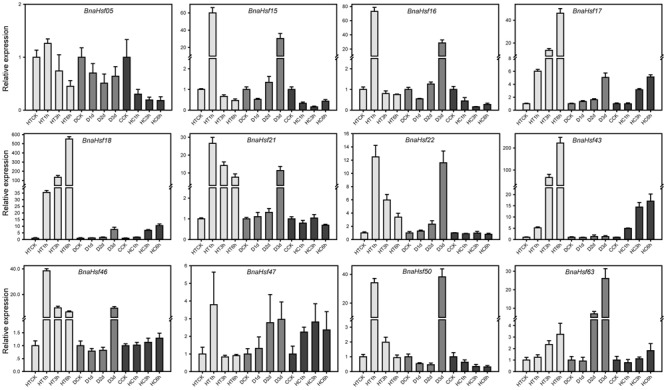
Real-time PCR analysis of the selected 12 representative *BnaHsf* genes responded to drought, heat and high CO_2_ treatments. HT, heat; D, drought; HC, High CO_2_.

The results of the qRT-PCR analyses were consistent with the expression patterns of selected *BnaHsfs* from RNA-seq data, and provided more details under progressive stresses (**Figure 6** and Supplementary Table 4). The RNA-seq data showed that transcription levels of *BnaHsf15*-*16* were decreased after exposure to 3 h heat (**Figure 5**), while in qRT-PCR results they were upregulated after 1 h of heat, and their expression then dropped to lower than basic levels (**Figure 6**). However, the strong induction of *BnaHsf15*-*16* began with a marked reduction in transcription after 1 day of treatment under drought stress. In contrast to *BnaHsf15*-*16*, the expression levels of *BnaHsf17*-*18* and *BnaHsf43* were progressively induced by all prolonged drought, heat, and high CO_2_ stress conditions. A similar pattern was found in *BnaHsf43* under drought and heat conditions. For *BnaHsf21*-*22*, the induction in their transcription was weakened as heat stress processed, while under progressive drought, the induction by stress showed an opposite enhancement (**Figure 6**).

### Regulatory *Cis*-Element Analysis of *BnaHsf* Genes

To identify the presence of putative regulatory *cis*-acting elements enriched in *BnHsf* genes, the promoter sequences upstream of their CDS were extracted and searched against the PLACE database (Higo et al., 1999). Analysis showed that HSEs were the most abundant *cis*-elements in promoter regions of *BnHsf* genes, including perfect type and active HSE variants (**Table 2**). Many of the subclasses of the *BnHsf* gene family possess the two types of HSE, although these are not present in subclasses A3 and A6A. Subclass A6A contains only one member, *BnHsf29*, which may be a pseudogene since its transcription cannot be detected in any tissues of rapeseed plants. The two members (*BnHsf17* and -*18*) of subclass A3 were found to have three other major types of stress-related *cis*-elements present in the *BnHsf* family, STRE, DRE/CRT, and MYCATRD22. The STRE element was first found to be stress responsive in yeast and can serve as a specific binding site for *HsfA1a* in *Arabidopsis* (Martinez-Pastor et al., 1996; Haralampidis et al., 2002; Guo et al., 2008). STRE was present in most subclasses of *BnaHsf* except A1A, A6A, and A7. DRE/CRT and MYCATRD22, two types of *cis*-elements responsive to drought stress, also appeared in most *BnHsf* subclasses. Two other stress related *cis*-elements, ABREOSRAB21 and LTRE, were found in some of the *BnaHsf* family members. In addition, three CO_2_-responsive elements (CCRE1/2/3) (Ohno et al., 2012; Tanaka et al., 2016) were observed in all *BnHsf* subclasses except A1A/B/D, A4A, A5, A6B, A7B, and B2A. The presence of these stress related *cis*-elements is likely responsible for the regulative expression patterns of *BnHsf* genes under drought, heat, and high CO_2_ conditions. Moreover, some phytohormone responsive related *cis*-elements were enriched in promoter regions of *BnHsf* member genes, which may be involved in the stress acclimation response and development.

**Table 2:** Putative *cis*-elements enriched in the promoters of *BnaHsf* family genes.

Sequence	*BnaHSF* subclass (members)	Element/stimulus annotation
NGAANNTTCN	A1A (Hsf02), A1B (Hsf03), A1D (Hsf08),A1E (Hsf10/11/14), A2 (Hsf15/16), A4A (Hsf19 21/23/24), A4C (Hsf25), A5 (Hsf28), A6B (Hsf31/33/34), A7A (Hsf35 38), A7B (Hsf39), A8 (Hsf42), B1 (Hsf44 46), B2A (Hsf50), B2B (Hsf51 54), B3 (Hsf55), C (Hsf63)	Perfect HSEs; heat stress elements
NGA(/N)N(/A)NNT(/N)N(/T)CN	A1D (Hsf09), A2 (Hsf16), A4A (Hsf19/22/23), A6B (Hsf31/34), A7A (Hsf36/37), A8 (Hsf41), B1 (Hsf46), B2A (Hsf48 50), B2B (Hsf51/54), B4 (Hsf59/60), C (Hsf61/62)	Active HSE variants
AGGGG	A1B (Hsf03), A1D (Hsf08/09), A1E (Hsf13), A2 (Hsf16), A3 (Hsf17/18), A4A (Hsf19), A4C (Hsf26), A5 (Hsf28), A6B (Hsf30 32), A8 (Hsf43), B1 (Hsf45), B2A (Hsf48/49), B2B (Hsf53), B3 (Hsf55/56), C (Hsf64)	STRE; stress-responsive element
RYCGAC	A1A (Hsf02), A1D (Hsf08/09), A1E (Hsf11 13), A2 (Hsf15/16), A3 (Hsf17/18), A4A (Hsf19/20/22/23), A4C (Hsf26), A7A (Hsf35), A7B (Hsf40), B1 (Hsf44/45/47), B2A (Hsf50), B3 (Hsf56), B4 (Hsf57), C (Hsf64)	DRE/CRT; drought, cold
CACATG	A1A (Hsf02), A1B (Hsf05/06), A1D (Hsf07 09), A1E (Hsf14), A2 (Hsf15), A3 (Hsf17/18), A5 (Hsf27), A6B (Hsf34), A7A (Hsf36 38), A7B (Hsf40), A8 (Hsf41), B1 (Hsf45), B2B (Hsf53/54), B3 (Hsf56), B4 (Hsf59/60), C (Hsf61/62/63)	MYCATRD22; dehydration, ABA
ACGTSSSC	A1B (Hsf03/04/06), A2 (Hsf16), A3 (Hsf17), A6A (Hsf29), A6B (Hsf34), A7B (Hsf39/40), B2B (Hsf53)	ABREOSRAB21; ABA-responsive elements
TGACGT	A1E (Hsf11), A2 (Hsf16), A3 (Hsf18), A4C (Hsf26), A6A (Hsf29), A7A (Hsf37), A8 (Hsf41/42), B1 (Hsf47), B2B (Hsf53), C (Hsf61/62/63)	CCRE1; CO_2_-responsive element
ACGTCA	A1E (Hsf10 14), A2 (Hsf15/16), B1 (Hsf46), B2B (Hsf52), B3 (Hsf55), B4 (Hsf60), C (Hsf63/64)	CCRE2; CO_2_-responsive element
TGACGC	A3 (Hsf18), A7A (Hsf36)	CCRE3; CO_2_-responsive element
CCGAAA	A1D (Hsf07), A2 (Hsf15), A6B (Hsf34), A7A (Hsf37), A7B (Hsf39), B1 (Hsf45/47), B2A (Hsf49), B2B (Hsf52 54), B4 (Hsf57)	LTRE; low temperature responsive element
TAACAGA	A1B (Hsf03/06), A3 (Hsf17), A6A (Hsf29), A8 (Hsf43), B2A (Hsf50)	GARE1OSREP1; Gibberellin
GCCGCC	A1B (Hsf03/04), B3 (Hsf56), B4 (Hsf58), C (Hsf64)	GCCCORE; Jasmonic acid
AWTTCAAA	A1D (Hsf09), A1E (Hsf10/11), A4A (Hsf19/21), A4C (Hsf25/26), A5 (Hsf27/28), A6A (Hsf29), A6B (Hsf30 34), A7A (Hsf35 37), A8 (Hsf41 43), B1 (Hsf44/45), B2B (Hsf54), B3 (Hsf55), B4 (Hsf59), C (Hsf61/62)	ERELEE4; Ethylene; senescence

## Discussion

### High Number of *Hsf* Family Genes in Rapeseed Genome

*Brassica napus* (rapeseed, genome AACC) is an amphidiploid species formed by recent interspecific hybridization between ancestors of *B. oleracea* (genome CC) and *B. rapa* (genome AA) (Chalhoub et al., 2014). In this study, we identified 64 *Hsf* genes in the genome of *B. napus*. Unlike yeast and animals, plants usually have many Hsf coding genes. There are 21 *Hsf* member genes in the model plant *Arabidopsis thaliana* (Nover et al., 2001), 25 members in rice (Chauhan et al., 2011), 56 *Hsf* genes in wheat (Xue et al., 2014). To date, *BnaHsfs* represent the largest *Hsf* gene family in plant species of which *Hsf* member genes were analyzed. The diversification of plant *Hsfs* is presumably the result of gene- and whole-genome duplications (WGD) at different points in evolution, followed by gene loss (Chalhoub et al., 2014). In the case of rapeseed, the allopolyploid process, that followed from the fusion of genomes A and C, might also play a crucial role in the expansion of the *BnaHsf* gene family. In addition, the large size of the *BnaHsf* family may have been needed for adaptation of rapeseed to diverse climatic zones.

### Structural Analysis of *BnaHsf* Genes

Similar to other plant *Hsf* families, the modular structure of rapeseed Hsf proteins is well conserved. While in comparison with that in *Arabidopsis*, there is no *Hsf* member in subclass A9 subclass in rapeseed. This differs from other eudicot plants, most of whom have sub classA9 *Hsfs*. This subclass is also lost in the *Hsf* family of *B. rapa* (Huang et al., 2015). The DBD is characterized as a central domain for the Hsf protein: it specifically binds to HSEs in the target promoter region, and subsequently activates the transcription of associated heat-inducible genes. The DBD domain of plant *Hsfs* is encoded by two regions separated by an evolutionarily conserved intron, which was inserted immediately adjacent to the HTH DNA binding motif (Nover et al., 2001; Scharf et al., 2012). Most *BnaHsf* genes have this intron in their DBD domains; however, no intron was found in *BnaHsf63* and -*64* genes from class C, as shown by their gene structure (**Figure 3A**). As far as we know, the fact that is this highly conserved intron in not present in the DBD domain of a plant *Hsf* is unique. Furthermore, this fact may indicate that *BnaHsf63* and -*64* have a novel regulation pattern relative to other *Hsf* genes.

### Diverse Transcriptional Patterns of *BnaHsf* Family Genes during Development and Abiotic Stresses

The functional diversification of *BnaHsf* family genes was also found in the tempo-spatial expression profile of these genes during development and abiotic stress treatments. Among the tissues at different developmental stages, subclasses A1A, A2/3, A4C, A5, A8, and B2 were found to be constitutively expressed at relatively high levels in all the tissues examined. While almost all member genes from subclasses A6/7 and B3/4 were hardly detected in any tissue, *BnaHsf35* from subclass A7A showed a high level of expression in root tissue. Subclasses B1 and B2A also showed high levels of expression in root tissue. Members of class C also showed increased expression in root tissue, but were not expressed in ovule tissue. These results suggest that these *BnaHsf* genes may be involved in root development. Under abiotic stress, many *BnaHsf* genes were upregulated in response to drought treatment. The number of drought induced *BnaHsf* genes was comparable to those induced by heat. This suggests that *Hsf* genes may also play an important role in the response and the acclimation to drought stress in rapeseed. Furthermore, the most inducible *BnaHsf* genes were upregulated by both drought and heat treatment, as shown by the combination of RNA-seq and qRT-PCR data. While *BnaHsf43* of subclass A8 was only induced by heat, *BnaHsf07* of subclass A1D and members of class C were predominantly upregulated by drought.

According to the combined transcriptional analysis, heat inducible *BnaHsf* genes could be divided into three groups. The first group consisted of *BnaHsf15* and -*16* (subclass A2), *BnaHsf47* (subclass B1) and *BnaHsf50* (subclass B2A), in which the expression of member genes exhibited an immediate and strong induction after 1 h of heat to a high level of 40∼70-fold of that in non-stressed control, followed by a dramatic drop to the basic expression level after 3 and 6 h of heat treatment, even was slightly suppressed in subclass A2 after 3 and 6 h of heat. It may be that this group of genes governs early heat stress response. The second group contains *BnaHsf21* and -*22* of A4A, and *BnaHsf46* of subclass B1. The transcription of the second group members showed also a fast and strong upregulation after 1 h of heat exposure. This upregulation of gene expression gradually declined after 3 and 6 h of heat, but still maintained at a high level relative to that in control. Genes from the second group might be involved in both early and late heat response. The last group greatly differed from the other two, comprised of *BnaHsf17* and -*18* of subclass A3 and *BnaHsf43* of subclass A8. The genes from this third group were upregulated after 1 h of heat, and this induction was continuously enhanced as the stress treatment proceeded, finally peaking after 6 h of heat stress (46∼550-fold vs. control). The members of this group likely have some function to facilitate acclimation to prolonged heat stress.

Drought induced *BnaHsf* genes seemed to have a single expression pattern. The genes continuously increased transcription during exposure to drought, and reached peak expression after the 3 days drought treatment. *BnaHsf* genes also played an important role in response to high CO_2_ treatment, as *BnaHsf18* (A3), *BnaHsf21* (A4A) and *BnaHsf43* (A8) were strongly upregulated, while members of A2 and B2 subclasses were downregulated.

### Various Regulatory *Cis*-Elements Enriched in the Promoters of *BnaHsf* Genes

Regulatory element analysis revealed that there were many stress-related *cis*-acting elements enriched in the promoter regions of *BnaHsf* family genes. HSEs were found to be the dominant *cis*-elements (**Table 2**). Complex interactions may exist among *BnaHsf* genes, and these may come about via *trans*-acting regulation, as HSEs are marker binding sites for plant Hsf proteins. Previous work has supported this idea that *HsfA1a*/*b* target class B *Hsf* genes and are responsible for their induction during heat response in Arabidopsis (Lohmann et al., 2004; von Koskull-Döring et al., 2007), and that *HsfA5* inhibits the activity of *HsfA4* (Baniwal et al., 2007). Other abiotic stress-related *cis*-elements, including STRE, DRE/CRT, MYCATRD22, ABRE, CCRE, and LTRE were also major regulatory elements found in *BnaHsf* genes. The presence of these stress-related elements seemed to be correlated to the expression response of *BnaHsfs* to heat, drought, and high CO_2_ treatments. For example, many drought related DRE/CRT and MYCATRD22 elements found upstream were associated with a marked induction of *BnaHsf* genes by drought stress. The two CCRE elements situated in promoter region also agreed with our observation of high induction levels of *BnaHsf18* under high CO_2_ treatment. Unlike *BnHsf15* and -*16* of subclass A2, the heat highly inducible genes *BnHsf17* and -*18* of subclass A3 do not have functional HSE elements, but rather an STRE element was found upstream of the target genes. The STRE element was identified to be stress responsive, and serves as a direct binding site for HsfA1a besides HSE in *Arabidopsis* (Guo et al., 2008). Furthermore, the deletion of STRE from the promoter of the Arabidopsis *Hsp90-1* gene decreased its promoter activity under heat stress conditions (Haralampidis et al., 2002). These findings suggest that STRE also plays a crucial role in transcriptional regulation under heat conditions, as do HSEs. Moreover, the different heat induced expression patterns of subclass A3 and subclass A2 *BnaHsf* genes provides evidence for differential transcription regulation abilities of STRE and HSE element. Unexpectedly, rapeseed subclass A1A was not heat inducible, although HSE elements are found in the promoter. While *HsfA1a* serves as master regulator of thermotolerance in tomato (Mishra et al., 2002), it also functions actively in *Arabidopsis* under heat stress. These results may indicate differential gene regulation of rapeseed *Hsf* genes from those found in other plants, even those in the Brassicaceae.

## Conclusion

Our genome-wide investigation of *Hsf* genes in *B. napus* reveals the largest plant *Hsf* gene family to date. With expression profile analysis, novel members of *BnaHsf* family were found to respond to high temperatures, as well as drought and high CO_2_ stresses. Further characterization of these novel multifunctional *BnaHsf* genes will improve our understanding of the acclimation response of plants to multifactorial and combinational abiotic stresses, and may also provide useful genetic resources for further research on abiotic stress resistance in crops.

## Author Contributions

XZ and WH conceived and designed the research. XZ, CH, LZ, HL, JY, and ZH performed the experiments and bioinformatics. XZ and CH analyzed the data. XZ and CH wrote the manuscript. All authors read and approved the final manuscript.

## Conflict of Interest Statement

The authors declare that the research was conducted in the absence of any commercial or financial relationships that could be construed as a potential conflict of interest.

## Footnotes

1 http://www.genoscope.cns.fr/brassicanapus/

2 http://cn.expasy.org/tools

3 http://meme-suite.org/tools/meme

4 http://gsds.cbi.pku.edu.cn
