# Supplementary material for: Systematic Analysis of Hsf Family Genes in the Brassica napus Genome Reveals Novel Responses to Heat, Drought and High CO2 Stresses
Source: Front Plant Sci. 2017 Jul 6;8:1174. doi: 10.3389/fpls.2017.01174 (PMC5498556; doi:10.3389/fpls.2017.01174)
Supplement: Supplementary file 5 [file Supplementary_Captions.DOCX]

Supplementary Material

Systematic analysis of *Hsf* family genes in the *Brassica napus* genome reveals novel responses to heat, drought and high CO_2_ stresses

Xiaoyi Zhu^†^, Chunqian Huang^†^, Liang Zhang, Hongfang Liu, Jinhui Yu, Zhiyong Hu, Wei Hua*

*** Correspondence:** Wei Hua, Email: huawei@oilcrops.cn

**Supplementary Figures and Tables**

**Supplementary Figure legends**

**Supplementary Figure 1.** Motif sequences identified using MEME tools in rapeseed *Hsf* genes.

**Supplementary Table legends**

**Supplementary Table 1.** Primers used in the study.

**Supplementary Table 2.** RNA-seq data of expression levels of *BnaHsf* family genes across different tissues and organs.

**Supplementary Table 3.** RNA-seq data of expression levels of *BnaHsf* family genes under heat, drought, and high CO_2_ treatments.

**Supplementary Table 4.** Quantitative real-time PCR data of the selected 12 representative BnaHsf genes responded to drought, heat and high CO2 treatments.

**Supplementary Datasheet 1.** Protein sequences of *BnaHsf* family genes with AtHsf genes used for phylogenetic analysis.
