# Supplementary material for: Systematic Analysis of Hsf Family Genes in the Brassica napus Genome Reveals Novel Responses to Heat, Drought and High CO2 Stresses
Source: Front Plant Sci. 2017 Jul 6;8:1174. doi: 10.3389/fpls.2017.01174 (PMC5498556; doi:10.3389/fpls.2017.01174)
Supplement: Supplementary file 6 [file Data_Sheet_1.DOCX]

**Supplementary Datasheet 1.** Protein sequences of BnaHsf family genes with AtHsf genes used for phylogenetic analysis.

>BnaA01g08640D (BnaHsf01)

MMDGVTGGDSKSGEATTAPPLRNPHPATLLGANALPPPFLSKTYDMVEDPASDAIVSWSPANNSFVVWDPPEFSRSLLPRYFKHNNFSSFVRQLNTYGFRKVDPDRWEFANEGFLRGQKHLLKTISRRKSTHGHGTSSSNTQSHQGHMASLSSCVEVGKFGLEEEVEQLKRDKNVLMQELVKLRQQQQSTDSKLQTMVKSLQTMEQRQQQIMSFLAKAVQNPTFLSQFIQKQTDSNNMHVTEASKKRRLTEDGTTAASDGQIVKYQPIRSDSMMWNMMNTDEKFPFSPNRDSGVTFQEVLLPTTSGQSQAYAPIPSASTSLMPMIPPMPQESISDSPTENYMNAEKDVSEAFISPSPFLDGGSVPNQLEGLPQDLDIDELMSNCDIFEEYLAQSPVFGDETTLESSDANGGHVDKLIEELGHLTSETKQL

>BnaA01g09690D (BnaHsf19)

MDESNHGGSSSSLPPFLTKTYEMVDDSSSDSIVSWSQSNRSFIVWNPPEFSRDLLPRFFKHNNFSSFIRQLNTYGFRKADPEQWEFANDDFVRGQPHLMKNIHRRKPVHSHSLPNLQAQQNALTDSERQRMSSQIERLTKEKEGLLQKLHRQEQERDVFEQQVKKLKDQLQHMEKRQKTMDSYVSQVMETPELALNLSPCLPETNERKRRLPRMLEDNQACVVVREEGSTSASDETEHQVEELESSIAVWENLVSVSDDSCEGMAQSTGSMMILDVDDSSTCPESPPLSCIHLSIDTCPKSPPSHKIIDINSDVSKEQNIVAPAPPPPATGVNDVFWQQLLTENPGSAEQREVQPEKKEDKGEDPKIKLGTARININAPFLDNPYLSPHHLASLTQSTPCPPSLHSSAAATASQDSTLLVVLTSSHRLLQTRLRPWSLRHLGDMHKIVSLTISDANLSSTPISHSSIYPKNPPTLITQLSNFEALKLSSETISHEIPNNTSYLISLKNLFLRCHSRS

>BnaA01g24460D (BnaHsf32)

MDPSYRFIKEEFPRGFNDSPSPPSSYLYTTSMAPNDPTTTLSSPQPIEGLHESGPPPFLTKTYDLVEDSRTNHVVSWSQANNSFIVWDPESFSMTLLPIFFKHNNFSSFVRQLNTYGFRKVNPDRWEFANEGFLRGQKHLLKTIRRRKTSNNNQMQPPQSSSQLQSLDNCCIEVGKYGLDGEMDSLRRDKQVLMMELVKVRQQQQSTKMDLTLLEDKLKKTESKQKQMMSFLARAMQNPDFLQQLIEQKEKRKNTEEAIDKKRQRPIDQGKRHDAGSSSAFFDMKQETYGDMSELDRLAMHIQGLGDQCNKEDVVLDVGKGNEEEQHKERYQDENNEIYGEGFWEDLLNEGQNFDLQGDDEENVDVLIEQLGYLGSSRH

>BnaA02g03270D (BnaHsf03)

MESVQSAPSANSNSPSIPPPVSSVPPFLSKTYDMVDDPSTNDVVSWSSGNNSFVVWNVPEFSKVLLPKHFKHNNFSSFVRQLNTYGFRKVDPDQWEFANEGFLRGQKQLLKGIVRRKPQNQQQTQVQNSSVSACVEVGKFGIEEEVERLKRDKNVLMQELVRLRQQQQATEHQLQNVGQKVQVMEQRQQQTMSFLAKAVQSPGFLNQLVQQNNDGNKHIQGSNKKRRLPGDEEESCGELNRQIVKYQPSINEAAQTMLRQVLNRSSSPVSNNPESFLLSDAPSSNSPNSAHSAMNQVHEAGLVDHLQAGPSPSQGGVAAAASWSPESELVGLETGDGVCFDPIMAALGGSLEIESDEVSPVGEGEMTELQNEAPKLPGVQDSFWEQFFADETAVIGEGDVIISEAVENCEMVMEQEPKEWKQQEMNHLTEQMELLSSEAQRK

>BnaA02g22350D (BnaHsf29)

MDYNLPTPLEVLRETGPTPFITKAYNIVEDSSTNNIVSWSRDNNSFIVWEPETFALICLPIYFKHNNFSSFVRQLNTYGFKKIDTERWEFANEYFLRGQKQLLKNIKRRKTSSQTEMLEKFGLEREIQGMRRDKAALVIELARLRQKQESVKTYLRFMEEKLIITERKQQMMMDFLLKKVKNPSFLENIKKRKQQEIENLEQSEEMTSSHGVEDYETFVKAEPEEYGDQFGGVFGNVDELHIASMEDQRQDDRMCILGEHLL

>BnaA03g19560D (BnaHsf55)

MEDDHLRCHDNINDDERLPLEFMTGKQTSTAELQPPQPPPFLVKTYKVVEDPTTDEVISWNEDGTGFIVWQPAEFARDLLPTLFKHCNFSSFVRQLNTYGFRKVSTTRWEFSNEMFRKGQRELMCNIRRRKSYPHSHSHNKSHQVVPTTTTEKQEDHHLEDQRSSSSSSPFVYSALLDENKCLKNENEFLSSELGKTKKKCKQLMELVERYKGEENNDDDDDDDDNDDQGLKLFGVKLE

>BnaA03g22890D (BnaHsf15)

MSRSNSLLTLAQKQSQSFGLWIRNGQQLKVELEEETVTYGGSAAASSSVGSSSSPRPMEGLNETGPPPFLTKTYDMVEDPATDTVVSWSNGRNSFIVWDSHKFSTTLLPRFFKHSNFSSFIRQLNTYGFRKIDPDRWEFANEGFLAGQKHLLKSIKRRRNMGLQTVNQQGSGSGSGSGSGMSCVEVGQYGFEGEVERLKRDHSVLVAEVVRLRQQQHNSKSQVAEMEQRLLVTEKRQQQMMAFLAKALNNPNFVQQFALMSKEKKGLFGSDVGRKRRLTSSPSLGTIEERVLHDHMEFDRMKDDMETLLAAAIDDEASKDEQCLEAMNVMMEDGPLEPEIDVKVEDLVASPLDWGSEDLHDIVDQMGFLGSEP

>BnaA03g24840D (BnaHsf51)

MPGEQTGEVGGGCSAGNSGGSSGGGGGGEPQRSIPTPFLTKTYQLVDDPVYDELISWNEDGSTFIVWKAAEFARDLLPKYFKHNNFSSFVRQLNTYGFRKLVPDRWEFSNDCFRRGEKILLRDIQRRKISLPAAAAAPVLAHVLSPSNSGEEQVISSNSSPAGTGGGGGGGSVGVAPQRTTSCTTAPELMEENERLRKENVQLSQELTKLKGLYSNIYKLMSNFTSGGADCLKPLDLMPERQEMSEEAIETGAGLKLLTPRLFGVSIGVKRARRDQELGAAAVEEDDNQEEEEQGSEDKSEPMEENNSVDHNGPWLELGK

>BnaA03g28190D (BnaHsf10)

MRSICESVPAANSSSASTTVVNSIPPFLSKTYDMVDDPLTDEVVSWSSGNNSFVVWNVPEFAKQFLPKYFKHNNFSSFVRQLNTYGFRKVDPERWEFANDGFVRGQKQLLKSIIRRKPSQVQPQKKPQVQHSSVGACVEVGKFGLEEEVERLQRDKNVLMQELVRLRQQQQGTEHHLQNVGQKVHVMEQRQQQMMSFLAKAVQSPGFLNQFSQQSSEGNQHISESNKKRRLPVEDQKNSGSHGVNGLSRQIVRYQSSVNESANSVLQQIQSMSSSRGCHEPLSNNHGSFLLGDVPNVSDNGSSSNGAYGVAFTDVSSNGAMKPHDPYAQADLLAPRQGAAAASGSSSSDLVGCETDNGECLDPMMVVLDESTILGRDGTINELLPGGEDSLWEQFFGESTETGNIDELVSGSMDNEMIMEQLGLQSNLRNVLRNNQQMNHLTEQMGLLTSDALRK

>BnaA03g37460D (BnaHsf63)

MEDKNNNTTSTNTNVIAPFVAKTYQMVNDPSTDWLITWGPAHNSFIVADPLDFSQRILPAYFKHNNFSSFVRQLNTYGFRKVDPDRWEFANEHFLRGQKHLLKNITRRKHARGMMDVEDGEIVKEIERLRDEQRELELEIQRMNQRIEATEKRPEQMMAFLYKVVDDPDLLPRMMLEKERLVSDKKKRRVTVKSEEGAEEEERVFGVVSPSPSPPENSIGWVVPMQRQGTFGSYYNETGMVANSMRSTSSKSSSLTSTLSLPESVNGGCGSVQGETRDKKAATFGGVVESNPQTPPYPFSLFRGGF

>BnaA03g41540D (BnaHsf36)

MNPFLHRENDPPPSPPKPVEGLHEIGPPPFLIKTFEIVEDPSTDHIVSWNRGGTSFVVWDLHSFSEFLLPRHFKHSNFSSFIRQLNTYGFRKIEAERWEFANEGFLLGQRHLLKSIKRRASFALSSSSIQDPCVELRKEKQLLLMELVSLRQQQETNKSYIKAMEQRIEGAERKQRKMMSFLARAMQSPSFLHQLLKQRDMRVKELEDEAAERGRGSSSVSELEALALEMQGYGKQKNVKEEEDMVVERELDDGFWEELLSNETPQHQITFIIHFQIQKNKQFPKMV

>BnaA03g41550D (BnaHsf37)

MNPFLHRENDPPPSPPKPVEGLHEIGPPPFLIKTFEIVEDPNTDHIVSWNRGGTSFVVWDLHSFSEFLLPRHFKHSNFSSFVRQLNTYGFRKIEAERWEFANEGFLLGQRHLLKSIKRRASFASSSIIQDPCVELRKEKQLLLMELASLRQQQQTARIYIKSMEQRIEGAEKKQRMMMSFLARAFQGPTFLYQLLQERDMRLKEVEVEVEERERGSSVSELEALALEMQGYGRQRNVKEEEDMVVERELDDGFWEELLSNESLASTSSS

>BnaA03g53750D (BnaHsf44)

MTAAQRTVPAPFLSKTYQLVDDHSTDGVVSWNEDGTAFVVWETAEFAKDLLPKYFKHNNFSSFIRQLNTYGFRKTVPDKWEFANDHFRRGQEDLLSEIRRRKAMIAAAAESNSAGDDDSTSSPGSKNPGSVENMVADLSGENKKLKRENDSLSSELAAAKRQRDELVAFLTDQLKVGPEQIDQMVKGGKFKPNITGDCSSEEESDCEGCGGDGVEKEGVGEGLKLFGVWVKGERKKRGRDEKNFVVGGSHRPDIKNVDFHAPLWKSSKVCN

>BnaA04g06390D (BnaHsf27)

MNGALGNSSASVNGGGGGDGAGGPAPFLVKTYEMVDDSSTDEIVSWSPNNNSFIVWNHAEFSRLLLPTYFKHNNFSSFIRQLNTYGFRKIDPERWEFSNDDFIKDQKHLLKNIHRRKPIHSHSQPPVSSADQERAALQEHMDKLSREKAAIEAKLFKFKQHKSTAKHNFDEMAEHVEDMEKRQEKLMGFLETAMRNPTFIKSFGRKVEELDLDVSAYNKKRRLPQGEQSKPPSEDSHLDNSSGSSRPESGNIFHQNFSSKLRLVLSPAVSDLNMVSHSIQSSNEGGASPKGILSGSDPKTTQTRREGLPFAPEALELADTGSCPGILLLNDKTIPEPLQQMITSSEETDGSFSCHLNLTLASAPLPDKTASQIAKTTHKSQEIGRVTELNFNSIETSSSEKNRGQQEVAAGGKQANAAPPARVNDVFWEQFLTERPGSFDNEEASSTYRENPCEEQEEKRNGHVMSRNTKNIEQLTL

>BnaA05g16880D (BnaHsf33)

MDPSYRFIKEEFPTGFIVSPSPPSSSTYSYLYSTSMVPNDPTTSSSPQPIEGLHESGPPPFLTKTYDLVEDSRTNHVVSWSQAKNSFIVWDPQAFSTTLLPRFFKHNNFSSFVRQLNTYGFRKVNPDRWEFANEGFLRGQKHLLKTIRRRKTSSNNQIQPPQSSSQEHSLDNCCIEVGRYGLDGEMDSLKRDKQVLVMELVKLRQQQQSTKVYLKLIEEKLKKTESKQQQMMSFLARAMQNPDFLQQLMEQKEKRKEMEEAISKKRQRPIDQGTRNVVTVEDYDDDDDGAYMKQEMYGDLSGFEMSELDSLAMHIQGLGDQFTREEVLDVQKRDEEEEQEGYPKVNNEIYGEGFWEDMLNEGQNFDFEGVEENVDVLIEQLGYLGSSSN

>BnaA05g32280D (BnaHsf12)

MGSNSESVSTVVSSIPPFLSKTYDMVDDPLTDEVVSWSSGNKSFVVWNVPEFARLFLPKYFKHNNFSSFVRQLNTYGFRKVDPDRWEFANEGFIRGQKQLLKSIVRRKPSQVQPPQQPQVQHSSVGACVEVGKFGLEEEVERLQRDKNVLTQELVRLRQQQQVTEHHLQNVGQKVHVMEQRQQQMMSFLAKAVQSPGFLNQFSQQNNEGNNQHVSEGNKKRRLPVEDQKYSGGYGANGLSRQIVRYQSSMNEAADTMLEQIHNMSNPQSRQEPLSNNHGSFLLGDVPNISDNGSSTNGGQFAVFSS

>BnaA06g21470D (BnaHsf48)

MVSDNGEPMMVITNESQRSVPTPFLIKTFNLVEDPSIDDVISWNEDGSSFVVWNPTDFARDLLPKHFKHNNFSSFVRQLNTYGFKKVVPDRWEFSNDFFRRGEKRLLKEIQRRKLTPQAAAVTPPPEVVAAAAAAQRVQTTKTVVSPSSSAEDQAVSPGGLSVELLEENEKLRSQNIQLNRELTQMKSLCDNIFSLMSNFSGSPPDQNCSPGPSSSHERMKPVEFFPAKRFSGETEVEEEASHRLFGVSIGLKRTRSEGVQVKAVGERSGGGGEKEETPWLRHYNRTNQRVCN

>BnaA07g05580D (BnaHsf61)

MEDDNNNNTNTNVIAPFVVKTYQMVNDPSTDWLITWGPAHNSFIVVDPLDFSQRILPAYFKHNNFSSFVRQLNTYGFRKVDPDRWEFANEHFLRGQKHLLKNIARRKHARGMVYGQEMEDGEIVREIERLKDEQRDLESEIQRMNQRIEATEKRPEQMMAFLYKVVEDPDLLPRMMLEKERTKLVSDKKKRRVTVKSEDEEENGRVFGIISPTPSPPENLYRTQSPENSMGWVVPMQKPGNFGSYNETGLISTSSTSSSLTSTLSLPESVNGGGGGCGSIQGETRYKEAATFGGVVESNPQTPPYPFSLFRGGF

>BnaA07g26740D (BnaHsf41)

MVKSSSDRVDSSSPSSSVAPFLRKCYEMVDDSSTDSIISWSTNGDNSFVISDTTVFSAQLLPKYFKHSNLSSFIRQLNIYGFRKVDADRCEFANDWFVRGQKELLKNVIRRKNVQSTSEHQSKTTTTTDASQEEKSGESELWKEVDILKGDKKALAQELVKVRQYQESTDTKMLHLEDRVQGMEESQQEMLSFLVMVMQNPSLLVQLLQPKENNWRKAEGGGAKILEEVTDEGETNSKGLPLVTYQAPSEGTTKSSSNEMNDFLRNADMLKFCLDENRVPLIIPDLYDDGAWEKLLLLSPSKKKKNKMQEKNVKKGIDDVTLEEKEEDEDGGRMELDKSLALELIEEEMEKADDFDFDIGQLTPERSKNLEILTQHMRLLASDQ

>BnaA08g04110D (BnaHsf57)

MAMMVENSYGYGSGGTGERIQLMVEGQGKAVPAPFLTKTYQLVDDPATDHVVSWGDDDTTFIVWRPPEFARDLLPKYFKHNNFSSFVRQLNTYGFRKIVPDRWEFANEFFKRGEKHLLCEIHRRKTSQMIPNQHSPFITHHPPPQIPFSGGASFFPLPPRVDAAAASMDERYWCESPPPRPRAIPTTVDAQVTALTEDNERLRRSNTVLMSELAHMKKLYNDIIYFVQNHVKPVAPSNSYLPSFLQKQQQPPPPLDYYNNATVPNHSPPTSQSSISVLENETNYKENNTRKTKLFGVSLPSTKKRSHHFSHQSSKTRLVLDNSDLGLNLMTASTR

>BnaA08g06910D (BnaHsf07)

MAGGNRSSTIDEAESGGGSVAMESKEIPQPQPVEVLSANAPPPFLSKTYDMVDDPATDPIVSWSSNNNSFLPTFIPSFSYLISADCLDSKFQWEFLSSFLVWTIRNKQLNLFGFRKVDLYRWEFANEVFLRVGNKHLLKTITRRKPAHVHGHGQQHSNGQNPSVSSCVEVGKFGLEEEVERLKRDKKVLMQELVRLRQQQQSTDNNLQRMVQRLQGMENRQQQLMSFLAKAVQSPHFLSQFLQQQNQQSESNGIVSSKDSASPDGQIVKYQPPMHEQAKAMLKKLMKMEPYKTGDDGFHLGDGTSTTTEMEISSNNVSGITLQEMPTASDIQSSSLTGATPENVTVAESPTPEEAIPSPDVLSLPEFASILPKKTTEMPSGNFMEPNMEDFSYFLDSDLFIDGDFPFDIDDIPSDPDLEPSVDKYASLLEDILMSSPLPDDMDVTPCLIDSLPPLDWIYITQDQMPSPMSHLASF

>BnaA08g08940D (BnaHsf21)

MDESNHGGSLPPFLTKTYEMVDDSSSDSIVSWSPQSNKSFIVWNPPEFSRDLLPKFFKHNNFASFIRQLNTYGFRKADPEQWEFANDDFVRGQPHLMKNIHRRKPVHSHSSPSLQPHPLTDSERQRMNDQIQRLTKEKEVLLEELHKQEKEREMFQQQVKELKDQLQHMEKRQKTMASFVSQVLEKPELALNLSPCLQETNERKRRFPRIGLEGSTSPSSQARELQVEQLESSIAVWENLVSEDSSESLGQETRSMMTLDVDESSTCPESPPLPCIQLSIDTCPTSPRTIDMNSEPDASKEQTIVAPPPPGAGGNDVFWQQLLTENPGSTGQKEVQSERKDDKAEKCWWDSRNVNTLTEQLGHLTS

>BnaA09g17760D (BnaHsf25)

MDENNGVSTSLPPFLTKTYEMVDDSSSDSIISWSDHNKSFIVKSPAEFSKDLLPRFFKHKNFSSFIRQLNTYGFRKVDPEKWEFANEDFVRGQPYLMKNIHRRKPVHSHSLQNLQAQSPLSESERQSMKDQIERLKREKEVLLADLQNQELERKGFELQVTALKDRLQHMEQRQRSIVTFVSQVLEKPGLSLNLETHERRKRKLQETETSLPPSRSHAAQVEKLESSLTFLENLVTESCDKGMDIDVNESTSCAESLSNGDNRAKSTKIDMNAEPVTIAVAAPKTGVNDVFWEQCLTENPGSIEQQE

>BnaA09g21510D (BnaHsf53)

MPGEQTGETPTAAGVGGGGGGCSAGNSGGSSGCGPVGGGDSQRSIPTPFLTKTYQLVDDPVYDDLISWNDDGSTFIVWRPAEFARDLLPKYFKHNNFSSFVRQLNTYGFRKVVPDRWEFSNDCFRRGEKILLRDIQRRKISQPAMAAAAAAAAAAAVNVAAAVPAVPHAVSPSNSGEEQVISSNSSPAAAAAASGGGGGQAGVVLQRTTSCTTAPELAEENERLRKENVQLSQELTKLKGLYSNIYTLMSNFTSGQTDCAPEGRALDLMPERMGEDMATASGVETGIGLKLDEDLTPRLFGVSIGAKRARRDEVVTAEEEDDERREGSNQEGGEQGSDVKSEPMEEDNSDEHNGPWLELGN

>BnaA09g24420D (BnaHsf08)

MDRGNSTKGGSVTMESTEIQPSPQPQPVAVLTGNAPPPFLSKTYDMVDDPATDSIVSWSANNNSFIVWDPPQFAKDLLPKNFKHNNFSSFVRQLNTYGFRKVDPDKWEFANEGFLRGQKHLLKTITRRKPAHGHGPQQSQHSIGQNSSVSSCVEVGKFGLKEEVERLKRDKNVLMQELVRLRQQQQSTDNQLQTMVQRLQGMENRQQQLMSFLAKAVQSPHFLSQFLQQQNQQNEGSRRISDTSKKRRFKRDGIVSNNKDSASPDGQIVKYQPPMHEQAKAMFSQLMKMEPYKAGDDGFLLGNGTSTTTSTEGTEMEISANNITGISLHEMPIASEIQSSSSPSGATPENVTVAEFPTPDEAIPSPDDLSLPEFADMLQENIAEVPPENFLETNMEDLSPILDSDLFNNDDLPFDIDDILRDPDLEPTVDDYASLFQDILMSSPVPDDMEVTPVDVTTKDGETEQKQMDNLTQQMGLLSPETIDLSR

>BnaA09g40360D (BnaHsf39)

MEPSSSSRAWSMPVPMKGLQEPGPCPFLTKTFEMVDDPNTNHIVSWNRGGISFVVWDPHSFSANILPLYFKHNNFSSFVRQLNTYFWRTSTKRSASSYCEMMGFRKIEAERWEFMNDGFLMGQRDLLKSIKRRTSSSAPPSLHHSQGDPCVELRQERHVLMMEISRLRQQEQRARGYIQAMEQRINGAEKKQRHMMSFLRRAVQNPALLQQLLEQQRKELEEASMDQVKPETVEHVSELEALALEMQGHGRQRTEEVEKELDDGFWEELLMNKDEGEEEDNVNTC

>BnaA10g05440D (BnaHsf59)

MAKMLENSYGYGNGERIQLMVEGQGKAVPAPFLTKTYQLVDDPATDHVVSWGDDDTTFIVWRPPEFARDLLPNYFKHNNFSSFVRQLNTYGFRKIVPDRWEFANEFFKRGEKHLLCEIHRRKTSQMIPNQHSPFITHHPPPQIPFSGGASFFPLPPRADASAAMDDHYWCESPSPRPRVVPTTADAQVAVLTEDNERLRRTNTVLMSELAHMKKLYNDIIYFVQNHVKPVAPSNSFLQKQQQPPPPPPPIDYYNNATVPNHSPPTSQSSITLLEDDTNPESITRKTKLFGVSLASSKKRSHHFSDQSSKTRLVLDKSDLGLNLMTASTR

>BnaA10g17670D (BnaHsf05)

MEPVLEPAPYVNSNPLPIPPPVNSVPPFLSKTYDMVDDPSTNEVVSWSSGNNSFVVWDVPEFSKVFLPKYFKHNNFSSFVRQLNTYGFRKVDPDRWEFANEGFLRDQKQLLKGITRRKSSHVQQQTQVQNSSLAPCVEVGKFGIAEEVERLKRDKNVLMHQQQATENQLKDAVQKVQAMEQRQQQMMSFLAKAVQSPGFLNQLVAQQSNDGNRQIQGSSNKKRRLPGEEANDVDRQIVKYQPSINEAAQTMLRQFLNMSSSPESESVPNNPGSFSPSSLNSSNTVSGVTLPEVSPDSAHSAAMNQVPEAGLAHHPQAGLVHPNLGPSPAAATWSPESECKTGNGECLDPIMAALGGSLEMEGDAVSPEGEGEVKELLVPQLPGVQDPFWEQFFADEAPVSGETDETILGSVENNDLAMEQELREWTRDQQQVNHITEQMGFLSSETHGGKNEKHVLCIIVLPFVFHRCIFFRGGCEGDMNGGGGDIS

>BnaA10g26390D (BnaHsf17)

MPLDILHGNPIPPFLSKTFDLVDDPSLDPVISWGLTGASFVVWDPVEFARIILPRNFKHNNFSSFVRQLNTYGFRKIDTDKWEFANEAFLRGQKHLLKNIHRRRSPQSNQACSSSITSQQSQGSPTEVGEEIEKLRKERRALMEEMVELQQQNRGTARHVDTVNQRLKAAEQRQKQMLSFLAKLFQNPGFLDRLKSLKGEGGGLGFEKARKKFIKHSPTGGEIVKYEADDWERLLMSEEKDTENILPSNQGMTSTDPKGKNLMNPSEEETMNQDYFLPFPTPEGLIKQEETWSMGFDTTIPSFSNEDVWGNTMDYNVAEFGSVPETSSGGCLPDVCWEQFAAGMTETGFNWPPGDDTTPMDDP

>BnaC01g01790D (BnaHsf46)

MMAAQRSVPAPFLSKTYQLVDDQSTDDVVSWNEDGSAFVVWKTAEFAKDLLPQYFKHNNFSSFIRQLNTYGFRKTVPDKWEFANDNFRRGQEELLSEIRRRKSVISAAGKCIVVGSPSESNSAGDDHGSSSTSSPGSKHPGSVENMVADLSGENEKLKRENSSLSSELAAAKRQRDELVAFLTEQMKVGPEQIDLMIKGGKKLKPAVEEEESDCEGCGGDGGAAVGEEKGVVGEGLKLFGVWVKGERKKRGRDEKNFLVGGSHMTEVKNVDFHAPLWKSSKVCN

>BnaC01g11370D (BnaHsf20)

MDESNHGGSSSSLPPFLTKTYEMVDDSSSDSIVSWSRSNRSFIVWNPPEFSRDLLPRFFKHNNFSSFIRQLNTYGFRKADPEQWEFANDDFVRGQPHLMKNIHRRKPVHSHSLPNLQSQQNPLTDSERLRMSSQIERLTKEKEGLLQKLHKQEQERDVFEQHVKTLKDQLQHMEKRQKTMDSYVSQVMETPELALNLSPCLPDTNERKRRLPRMLEDNRTCVVVREEGSTSASDETEHQVEQLEVWENLVSVSDDSCEGMAQSTRSMMILDVDESSTCPQSPPLSCIQLSIDTCPKSPPVKIMDMNSEPDVSEEQNIVAPPPPPAAGVNDVFWQQFLTENPGSTEQREVQPERKEDKGEDRSEKCWWTSRNVNAITEQLGHLTS

>BnaC02g06880D (BnaHsf04)

MEPVQSVPSENSNPPSIPPPVSSVPPFLSKTYDMVDDPSTNEVVSWSSGNNSFVVWNVPEFSKVLLPKHFKHNNFSSFVRQLNTYGFRKVDPDQWEFANEGFLRGQKQLLKGIVRRKPQNQQQTQVKNSSAGACVEVGKFGIEEEVERLKHDKNVLMQELVRLSQQQQATEHQLQNVGQKVQVMEQRQQQTMSFLAKAVQSPGFLNQLVQQNSDCNKHIQGSNKKRRLPGDEEESCGELNRQIVKYQPSLNEAAQTMLGQILNRSSSTRCESVSNNPDSFLLDDAHSTNSAHSTMNQVHEAGLVDHPQAVPSPSQGVAAAASSWSPESELVGFETGDGVCFDPIMAALGGSLEIESDEVSRVGEGEMTELQNEVPKLPGVQDSLWEQFFADEIGEGDDILSGAVENGDMVMEQEPNEWKQQEMNHLSEQMELLSSETRN

>BnaC02g17710D (BnaHsf43)

MVKSSDGVSSSSSSVAPFLRKCYEMVDDSSTDSIISWSSSADNSFVISDTNVFSAQLLPKYFKHSNLSSFVRQLNIYGFRKVDWEFANDWFVRGRKDLLKNVIRRKNVQSSDHQSKSVSKKQESTCKEDDTEKSELWKEVDILKGDKKALAQELVKVRQYQEVTDTKMLHLEDRVQGMEESQQEMLSFLVMVMQNPSLLVQLLQPKESSWRKAEGGGGGAKILEEVIDEGESDSSGLPLVTYQQPQSEGTAKSSSSDMNDFLRNADMLKFCLDENHVPLIIPDLYDDGAWEKLLLLSPSKKKNVKKGKDDATLKEEEEDETMKVIQEEMERADDFDFGQLTPERSRNLEILTQQIALLASNE

>BnaC03g23450D (BnaHsf56)

MEDDHLRCHDNINDEERLPLEFMTGKQTSTAELQPSQRPPFLVKTYKVVEDPTTDEVISWNGDGTGFIVWQPAEFARDLLPTLFKHCNFSSFVRQLNTYGFRKVSTTRWEFGNEMFRKGQRELMCNIRRRKSYPHSHSHNKSHQVVPTTTTENLEDHHLQDQRSSSVVYSALLDENKCLKNENEFLSSELGKTKKKCKQLMELVERYRGEEDDDETTTIRGLSCLE

>BnaC03g26940D (BnaHsf16)

MEQLKVELEEETVTYGGSAAASSSVGSSSSPRPMEGLNETGPPPFLTKTYDMVEDPATDTVVSWSSGRNSFIVWDSHKFSTTLLPRYFKHSNFSSFIRQLNTYGFRKIDPDRWEFANEGFLAGQKHLLKSIKRRRNMGLQTVVNQQGSGSGMSCVEVGQYGFEGEVERLKRDHSLLVAEVVRLRQQQHNSKSQVAEMEQRLLVTEKRQQQMMTFLAKALNNPNFVQQFALMSKEKKGLFGSDVGRKRRLTSSPSLGTIEERVLHDQEFDRMKDDMETLLAAAIDDEASNLVAASKDEQCLEAMNVMMEDGSLEPEIDVKVEDLVASPLDWDSEDLHDIVDQMGFLGSEP

>BnaC03g33280D (BnaHsf11)

MRSICESVPAANSSSASTTVVSSIPPFLSKTYDMVDDPLTDEVVSWSSGNNSFVVWNVPEFAKQFLPKYFKHNNFSSFVRQLNTYGFRKIDPERWEFANEGFVRGQKHLLKSINRRKPSQVQPQKQPQVQHSSVGACVEVGKFGLEEEVERLQRDKNVLMQELVRLRQQQQGTEHHLQNVGQKVHVMEQRQQQMMSFLAKAVQSPGFLNQFSQQSSEGNQHISESNKKRRLPVENQKGSGSHGVNGLSRQIVRYQSSMNESANSVLQQIQSMSSSRGCHEPLSNNHGSFLLGDVPNLSANGSSSNGASGVAFSDVSSNAAMKHHDPYAQADLLAPRQGAVVASGSSSSDLVGCETDNGECLDPMMAVLDESMMLGSDGAINELLPGAGDSLWEQFFGESPGIGITDELVSGCRRWGLSFVELQQQKYNDQKEVVVYLCIKLSFLSMCD

>BnaC03g43990D (BnaHsf64)

MEDDKNNNTSSTNTNVIAPFVAKTYQMVNDPSTDWLITWGPAHNSFIVADPLDFSQRILPAYFKHNNFSSFVRQLNTYGFRKVDPDRWEFANEHFLRGQKHLLKNIARRKHARGMTDAEDGEIVKEIERLRDEQRELELEIQRMNQRIEATEKRPEQMMAFLYKVADDPDFLPRMMLEKERTKQLVSDKKKRRVTVKSEEGAEEEERVFGVVSPSPSPSPENSIGWVVPMQRQGTFGSYYNETGMVTNSMISTSSKSSSLTSTLSLLESVNRGGGGGCGSIQGETRDKEAATFGGVVESNPQTPPYPFSLFRGGF

>BnaC03g52080D (BnaHsf49)

MVSDNGDPMMVITNESQRTVPTPFLIKTFNLVEDPSIDDVISWNEDGSSFVVWNPTDFARDLLPKHFKHNNFSSFVRQLNTYGFKKVVPDRWEFSNDFFRRGEKRLLKEIQRRKLTPQAAVTPPPEVVAAAAAQRVQTTKTVVSPSSSAEDQAVSPSWYCQTGNGGGLSVELLEENEKLRSQNIQLNRELTQMKSLCDNIFSLMSNFSGSPPERSSSPGASTSHDAMKTVEFLPAKRFSVETEGEEEASHRLFGVSIGLKRTRSEGVQVKAVGERPGGGGEKEETPWLRHYNRTNQRVCN

>BnaC03g62890D (BnaHsf22)

MDESNHGGSLPPFLTKTYEMVDDSSSDSIVSWSQSNKSFIVWNPPEFSRDLLPKFFKHNNFASFIRQLNTYGFRKADPEQWEFANDDFVRGQPHLMKNIHRRKPVHSHSSPSLQPHPLTDSERQRMNDQIERLTKEKEVLLQELHKQEKEREMFQQQVKELKDQLQHMEKRQKTMVSFVSQVLEKPELALNLSPCLLETNERKRRFPRIGLEGSTSPSSQARELQVEQLESSIAVWENLVSEDSSESLGQEKRSMMTLDVDESSTCPESPPLPCIQLSIDTCPNCPTSPRTIDMNSEPDTSKEPPPAAGVNDVFWQQLLTENPGSTEQKEVQAERKDDKAEECWWDLRNVNTLTEQLGHLTS

>BnaC04g28450D (BnaHsf35)

MNPFQLQEGDPPPSPPKPVEGLHEVGPPPFLIKTFEIVEDPNTDHIVSWNRGGASFVVWDLHSFSTFLLPRHFKHSNFSSFIRQLKTYGFRKIESDRWEFANDGFLLGQRQLLKSIKRRASFGSSPSTHDPCTELRREKQLLMMELVCLRQQQQTTRSYIKAMEQRIEGAERKQRQMMSFLARAMRSPSFLHQLLKQRDMRLKELEDEAAERERGSSMSELEALALEMQGYGKERNVKEEEDMVVERELDDGFWEELLSNESLASTSS

>BnaC04g29180D (BnaHsf28)

MNGALGNSSASVTGGGGGDGAGGPAPFLVKTYEMVDDSSTDEIVSWSSNNNSFIVWNHAEFSCVLLPTYFKHNNFSSFIRQLNTYGFRKIDPERWEFSNDDFIKDQKHLLKNIHRRKPIHSHSQPPVSSVDQERAALQEQMDKLSREKAAIEAKLLKFEQHKSTAKHNLDEMAEHVEDMEKRQEKLMSFLETAMRNPTFIKNFGRKVEDLDVSAYNKKRRLPQGEQSKPPSEDSHLDNSSGSSRPESGNIFHQNFSNKLRLELSPAVSDMNMVSHSIQSSNEGGASPKGILSGSDPNTIQTRREGLPFAPEALELADTGSCPGILLLNDKTIPEPLQQMITSSEETDGSFSCHLNLTLASAPLPDKTASQIAKTTHKSQEIGRVTELNFNSIETSASEKNRGQQEVAAGGKLTNAAPPARVNDVFWEQFLTERPGSSDNEEASSTYRENPCEEQEEKRNGHVISRCTKNIEQLTL

>BnaC05g24440D (BnaHsf09)

MDRGNRTSTNGGEGGSVTMESTEIQPSPQPQPVAVLTGNAPPPFLSKTYDMVDDPATDSIVSWSANNNSFIVWDPPQFAKDLLPKNFKHNNFSSFVRQLNTYGFRKVDPDKWEFANEGFLRGQKHLLKTITRRKPAHGHGHQQSQHSIGQNSSVSSCVEVGKFGLKEEVERLKRDKNVLMQELVRLRQQQQSTDNQLQTMVQRLQGMENRQQQLMSFLAKAVQSPHFLSQFLQQQNQQNEGSRRISDTSKKRRFKRDGIVSNNKDSASPDGQIVKYQPPMHEQAKAMFSQLMKMEPYKAGDDGFLLGNSTTTTTSTEGTEMEISANNITGISLQEIPTASEIQSSSSPSGATPENVTVAEFPTPDEAIPSPDVLSLPEFADMLQENIAEVPPENFLETNMEDLSPILDSDLFNNDDLPFDIDDILRDPDLEPSVDDYASLFQDILMSSPVPDDMEVTPVDDRTKDSETEQKQMDNLTQQMGLLSPETIDLSR

>BnaC05g29680D (BnaHsf34)

MDPSYRFIKEEFPTGFIVSPSPPSSSTYSYLYSTSMAPNDPTTSSSPQPIEGLHESGPPPFLTKTYDLVEDSRTNHVVSWSQAKNSFIVWDPQAFSTSLLPRFFKHNNFSSFARQLNTYGFRKVNPDRWEFANEGFLRGQKHLLKTIRRRKTSNNNQIQPPQSSSQEHSLDNCCIEVGRYDLDGEMDSLKQDKQVLVMELVKLRHQQESTKVYLKLIEEKLKKTESKQQQMMSFLARAMQNPDFLQQLMEQKEKRKEMEEAISKKRQRPIDQGTRNVVTVEDYDDDDSAYMKQEMYGDLSGFEMPELDSLAMHIQGLGDQFTRKEVLDVEKRDEEGEQEGYQKVNNEIYGEGFWEDMLNEGQNFDFEGDEENVDVLIEQLGYLGSSSN

>BnaC06g00310D (BnaHsf60)

MATMLENSYGYDNGGTGERIQLMVEGQGKAVPAPFLTKTYQLVEDPATDHVVSWGDDDTTFIVWRPPEFARDLLPNYFKHNNFSSFVRQLNTYGFRKIVPDRWEFANEFFRRGEKHLLCEIHRRKTSQMIPNQHSPFITHHPPPQIPFSGGASFFPLPPRVDAATAAMDDHYWCESPSPRPRLVPTTLDPQVAVLTEDNERLRRTNTVLMSELAHMKKLYNDIIYFVQNHVKPVAPSNSFLQKQQQPPPLIDYYNNATVPNHSPPTSQSSITLLEDDTNHESFTRKTKLFGVSLASSKKRSHHFSDQSSKTRLVLDKSDLGLNLMTASTR

>BnaC06g29140D (BnaHsf42)

MVKSSDRVDSSSSSVAPFLRKCYEMVDDSSTDSIISWSTNGDNSFVISDTTVFSAQLLPKYFKHSNLSSFIRQLNIYGFRKVDADRCEFANDWFVRGQKELLKNVIRRKNVQSTSEHQSKTTTTDASQEKKSGESELWKEVDILKGDKKALAQELVKVRQYQESTDTKMLHLEDRVQGMEESQQEMLSFLVMVMQNPSLLVQLLQPKENNWRKAEGGGGGGGAKILEEVTDEGETNSKGLPLVTYQKTPSEGAAKSSSNEINDFLRNADMLKFCLDENRVPLIIPDLYDDGAWEKLLLLSPSKKKKNKVQEKNVNDDVTLHEEDEDGGRMELDKSLALELIEEEMEKADDFDFDIGQLTPEKSKNLEILTQHMRLLASDQ

>BnaC07g07130D (BnaHsf62)

MEDDNNNNTNTNVIAPFVVKTYQMVNDPSTDWLITWGPAHNSFIVVDPLDFSQRILPAYFKHNNFSSFVRQLNTYGFRKVDPDRWEFANEHFLRGQKHLLKNIARRKHARGVIYGQEMEDGEIVREIERLKDEQRDLELEIQRMNQRIEATEKRPEQMMAFLYKVVEDPDLLPRMMLEKERTKLVSDKKKRRVTVKSEDEEENGRVFGIISPSPSPPENVYRTQPPENSVRWVVPMQKPGNFGSYNETGLISTSSTSSSLTSTLSLPESVNGDGGGCGSIQGETRYREAATFGGVVESSPQTPPYPFSLFRGGF

>BnaC07g32600D (BnaHsf38)

MNPFLHRENDPPLSPPKPVEGLHEIGPPPFLIKTFEIVEDPNTDHIVSWNRGGTSFVVWDLHSFSEFLLPRHFKHSNFSSFVRQLNTYGFRKIEAERWEFANEGFLLGQRHLLKSIKRRASFASSIQDPCVQLLKEKQLLSMELASLRQQQQTARIYIKSMEQRIEGAEKKQRMMMSFLARALQGPTFLYQLLQERDMRFKEVEVEERERGSSVSELEALALEMQGYGRQRNVKEEEDMVVERELDDGFWEELLSNESLASTSIL

>BnaC07g35520D (BnaHsf23)

MDESSHGGSSSTSLPPFLTKTYEMVDDSSSDSIVSWSQSNKSFIVWNPPEFSRDLLPKFFKHNNFSSFIRQLNTYGFRKADPEQWEFANDDFVRGQPHLMKNIHRRKPVHSHSLPNLQSQQNPLTDSERQRMNSQIERLTTEKQVLLEELHKHEEERELFEQQVKKLKDQLHHMEKRQRTMVSSVSQVLEKPELALNLSPCLPEANERKRRFPRVGLETMLEENHQTCGAVREEGSTSTSSHDATECQVERLESSIAIWENLVSDSCESMAQQSTRNMMTLDVDESSTCPESPPLSCIQLSIDIRLKSPPSPRTIDMNSEPDVSKEQNVVPPDPSPPAVGANDVFWQQLLTENPGSTEQREVQSEKAEDRSEKCWWNSRNVNTITEQLGHLTS

>BnaC08g04780D (BnaHsf58)

MAMMVENSYGYGSGGTGERIQFMVEGQGKAVPAPFLTKTYQLVDDPATDHVVSWGDDDTTFIVWRPPEFARDLLPKYFKHNNFSSFVRQLNTYGFRKIVPDRWEFANEFFKRGEKHLLCEIHRRKTSQMIPNQHSPFITHRPPPQIPFSDGTSFFPLPPRVDAAAASIDDRYWCESPPPRPRAIPTTVDAQVTALTEDNERLRRSNTVLMSELAHMKKLYNDIIYFVQNHVKPVAPSNSYLPSFLQKQQQPPPPLDYYNNATVPNHSPPTSQSSISVLENETNYKENNTRKTKLFGVSLPSSKKRLHHFSHQSSKTRLVLDNSDLGLNLMTAFTR

>BnaC08g32790D (BnaHsf40)

MEPSSSSRAWSMPVPMGGLQEPGPYPFLTKTFEMVDDPNTNHIVSWNRGGISFVVWDPRSFSATILPLYFKHNNFSSFVRQLNTYFWRTSTKRSASSYCEMGFRKIEAERWEFMNDGFLMGQRDLLKSIKRRTSSSAPPSLHHAQGDPCVELRQERHVLMMEISRLRQQEQRARGYIQTMEQRINGAEKKQRHMMSFLRRAVQNPALLQQLLEQQRKELEEASMDQVKPETVEHVSELEALALEMQGHGRQRTEEVERELDDGFWEELLIMNKDEDGVEANVNAC

>BnaC09g18620D (BnaHsf26)

MDENNGVSTSLPPFLTKTYEMVDDSSSDSIISWSDHNKSFIVKSPAEFSKDLLPRFFKHKNFSSFIRQLNTYGFRKVDPEKWEFANEDFVRGQPYLMKNIHRRKPVHSHSLQNLQAQSPLSESERQSMKDQIERLKRDKEVLLTDLQNQELERKGFELQVTALKDRLQHMEQRQRSIVTFVSQVLEKPGFSLNLETHETRKRKLQETETSLPPGRSHADQVEKLESSLTFLENLVTESCDKGMDIDVNESTSCAESLSNGDNRAKSTKIDMNAEPVTVAVAAPKTGVNDVFWEQCLTENPGSIEQQEVQSERRDVDANKIGDGRTFWWKAKNVNDIAERA

>BnaC09g41040D (BnaHsf06)

MEPVLEPAPSVNSNPSPIPPPINSVPPFLSKTYDMVDDLSTNEVVSWSSGNNSFVVWDVPEFSKVFLPKYFKHNNFSSFVRQLNTYGFRKVDPDRWEFANEGFLRDQKQLLKGITRRKSSHVQQQNQQQIQVQNSSLAPCVEELVRLRHQQQATEHQLKDAVQKVQAMEQRQQQMMSFLAKVVQSPGFLNQAQQSSNDGDRQIQGSSNKKRRLPGEEANDVDRQIVKYQPSINEAAQTMLRQFLNMSSSNECESVSNSLNSSNAVSGVTLPEVSPDSAHSAMNQVPEAGLAHHPQAGLVQPNLGPSPAAASWSPESECKTGNGECLDPIMAALGGSLEMEGDAVSPKGEGEMKELLVPQLPGVQDPFWEQFFADEAPVSGDAEETISGSVENNDLAMGQELSEWTRDQQQVNHITEQMGFLSSETHGGKNEKHLLCIDVLS

>BnaC03g73070D (BnaHsf52)

MPGEQTGEVGGGCSAGNSGGSSGGGGGESQRSIPTPFLTKTYQLVDDPVYDELISWNEDGSTFIVWKPAEFARDLLPKYFKHNNFSSFVRQLNTYGFRKVVPDRWEFSNDCFRRGEKILLRDIQRRKISQPAMAAAAAPVLAHVQVISSNSSPAGTGGGGGSVSVALQRTTSCTTAPELMEENERLRKENVQLSQELTKLKGLYSNIYKLMSNFTSGGADCAKTLDLMPERQEMSEEAIETGTGLKLLTPRLFGVSIGVKRARRDHELGAAAVEEDDNQEEEEQGSDDKSEPMEENNSVDHNGPWLELGK

>BnaC09g52680D (BnaHsf54)

MPGEQTGETPTAAGVGGGGGGCSAGNSGGSSGCGPVGGGDSQRSIPTPFLTKTYQLVDDPVYDDLISWNDDGSTFIVWRPAEFARDLLPKYFKHNNFSSFVRQLNTYGFRKVVPDRWEFSNDCFRRGEKILLRDIQRRKISQPAMAAAAAAAAAAAVTVAAAVPAVPHAVSPSNSGEEQVISSNSSPAAAAAASGGGGGQVGVVLQRTTSCTTAPELVEENERLRKENVQLSQELTKLKGLYSNIYTLMSNFTSGQADCAPEGKALDLMPERMGEDMATASGVETGIGLKLDEDLTPRLFGVSIGAKRARRDELVTAEEEDDERREGSNQEGGEQGSDVKSEPMEEDNSDEHNGPWLELGN

>BnaAnng15230D (BnaHsf14)

MGSISESAPTANSSTTVVMSSIPPFLSKTYDMVDDPSTDEVVSWSSGSNSFVVWNVPEFSKQFLPKYFKHNNFSSFVRQLNTYGFRKVDPDRWEFANEGFLKGQKQLLKSIIRRKPTQVQPPQQPQVQHSSVGACVEVGKFGLEEEVERLQRDKNVLMQELVRLRQQQQVTEHHLQHVGQKVHVMEQRQQQMMSFLAKAVQSPGFLNQFSQQSNNEGNQHISESNKKRRLPVEDQKNRGGSSQGLNGLSRQIVRYQSSMNESSNSMLQQIHNMSNTHTNNHGSFLLGDVPNPNLSDNGSSSNGPSGVVAFTDVSSNTTNQVLETNLPYPQPQADLLAPKQGAEGGSGSPSPDLVGGERDNGECLDPIMAVLDGSMMLETNELLPGVQDSLWEQFFGESSGIGDSDELVSGSVDNELIMEQLELQPNLRNVLSNNQQMNHLTEQMGLLTSDALRK

>BnaAnng31620D (BnaHsf24)

MDESSHGGSSSTSLPPFLTKTYEMVDDSSSDSIVSWSQSNKSFIVWNPPEFSRDLLPKFFKHNNFSSFIRQLNTYGFRKADPEQWEFANDDFVRGQPHLMKNIHRRKPVHSHSLPNLQPHPLTDSERQRMNDKIERLTKEKQVLLEELHKHEEERELFEQQVKKLKDQLHHMEKRQRTMVSSVSQVLEKPELALNLSPCLPEANERKRRFPRVVGLETMLEENHQTCGAVREEGSTSTSSHDATEHQVERLESSIAIWENLVSDSCESMEQQETRNMMTLDVDESSTCPESPPLSCIQLSIDIRLKSPPSPRTIDMNSEPDVSKELNTVSPTPPAVGANDVFWQQLLTENPGSTEQREVQSEKAEERSEKYWWNSRNVNTITEQLGHLTS

>BnaAnng36200D (BnaHsf47)

MMAAQRSVPAPFLSKTYQLVDDQSTDDVVSWNEDGSAFVVWKTAEFAKDLLPQYFKHNNFSSFIRQLNTYGFRKTVPDKWEFANDNFRRGQEELLSEIRRRKAVIAAAGKCVVVGSPSESNSAGDDHGSSSTSSPGSKHPGSVENMVADLSGENEKLKRENSSLSSELAAAKRQRDELVAFLTEQMKVGPEQIDQMIKGGGKKLKPAVEEEESDCEGCGGDNGGAAVEGEKGVVGEGLKLFGVWVKGERKKRGRDEKNFVVGGSHMTEIKNVDFHAPLWKSSKVCN

>BnaCnng02620D (BnaHsf18)

MSPEKDGVSIPTPLSIPISTRPESVPLHVDTDVASPLPMPLDILHGNPIPPFLSKTFDLVDDPSLDPVISWGPTGASFVVWDPVEFARIILPRNFKHNNFSSFVRQLNTYGFRKIDTDKWEFANEAFLRGQKHLLKNIHRRRSPQSNQACSSSITSHQSQGSPTEVGEEIEMLRKERRALMEEMVELQQQNRGTARHVDTVNQKLKAAEQRQKQMLSFLAKLFQNPGFLDRLKSLKGEGGGLGFKKARKKFIKHSPTGGEIVKYEADDWERLLMSEEEDTENILPSNQGMTSTDPKGKNLMNPSGEETMNQDYFLPFPTPEGLIKQEETWSMGFDTTIPSFSNEDVWGNTMDYNVAEFGSVPETSSGGCLPDVCWEQFAAGMTETGFNWPPGDDTTPMDDP

>BnaCnng06170D (BnaHsf13)

MRSNCESSTTVVSSIPPFLSKTYDMVDDPLTDEVVSWSSGNKSFVVWYVPEFSILFLPKYFKHNNFSSFVRQLNTYGFRKVDPDRWEFANEGFIRGQKQLLKSIVRRKPSQVQPPQQPQAQHSSRDKNVLTQELVGLRQQQQVTEHHLQNVGQKVHVMEQRQQQMMSFLAKAVQSPGFLNQFSQQNNEGNNQHVSEGNKKRRLPVEEQKYAGGYGANGLSCQIVRYQSSMNEAADTMLEQIHNMSNPHSRQEPLSNNHGSFLLGDVPNISDNGSSTNGASGVTLADVSSNPAINYHVPCEANQILEGSLPYAQVDLLAPNQGAAYGSSNSDVVGCETDNEECLDPIMAVLAGSMGLEANAVNELLPGVQDPLWEQFFGERPVIGDTEELVSGSVDNGLIMEQLELQSNLRNVLSSNQQMNHLTEQMGFLTSDALRK

>BnaCnng14280D (BnaHsf30)

MDPSYRFIKEEFPRGFNDSPSPPSSSTSSYLHSTSMAPNDPATLNSPQPIEGLHESGPPPFLTKTYDLVEDSRTNHVVSWSQANNSFIVWDQEYFSMTLLPIFFKHNNLSSFVRQLNTYGFRKVNPDRWEFANEGFLRGQKHLLKTIRRRKTNNCCIEVGKYGLDGEMDSLRRDKQVLMMELVKVRQQQQSTKMDLTLLEDKLKKTESKQKQMMSFLARAMQNPDFLQQLIEQKEKRKNTEEAIDKKRQRPIDQGKRHVVCVEDYDDGGGGYGRYGKDAGSSSAFFDMKQETYGDMSELDRLAMHIQGLGDQCNKEDVVLDVGKGNEEEQHKERYQDENNEIYGEGFWEDLLNEGQNFDLQGDDEENVDVLIEQLGYLGSSGH

>BnaCnng14290D (BnaHsf31)

MDPPYRFIKEEFPTGFNDSPSPPSSNLYTTSMAPNDPTTTLSSPQPIEGLHESGPPPFLTKTYDLVEDSRTNHVVSWSQANNSFIVWDQEYFSMTLLPIFFKHNNLSSFVRQLNTYVSTNLNTYVKLSRFYYTLHPLGFRKVNPDRWEFANEGFLRGQKHLLKTIRRRKTNNCCIEVGRRDKQVLMMELVKVRQQQQSTKMDLTLLEDKLKKTESKQKQMMRFLARAMQNPDFLQQLIEQKEKSKDTEEAIDKKRQRPIDQGKRHDAGSSSAFFDMKQETYGDMSELDRLAMHIQGLGDQCNKKDVVLDVGKGNEEQQHKERYQDENNEIYGEGFWEDLLNEGQNFDLQGDDEENVDVLIEQLGYLGSSGH

>BnaCnng36910D (BnaHsf02)

MMDGVTGGDSKSGEATTAPPLRNPHPATLLGTNALPPPFLSKTYDMVEDPASDAIVSWSPANNSFVVWDPPEFSRSLLPRYFKHNNFSSFVRQLNTYGFRKVDPDRWEFANEGFLRGQKHLLKTISRRKSTQGHGSSSSSNPQSHQGHMASLSSCVEVGKFGLEEEVEQLKRDKNVLMQELVKLRQQQQSTDSKLQSMVKSLQTMEQRQQQIMSFLAKAVQNPTFLSQFIQKQTDSGNMHVTEASKKRRLTEDGTTAAAAASDGQIVKYQPIRSDSTMSMMWNMMNTDEKFPFSPNRDSGVTFQEVLLPTTSGQSQAYAPISSASTSLMPMIPPMPQESISDSPTENYMNAEKDVSEAFISPSPFLDGGSVPNQLEGLPQDLDIDELMSNCDIFEEYLAQSPVFGDETTLESSDANGGHVDKLIEELGHLTSETKQL

>BnaCnng54110D (BnaHsf50)

MATNESQSQRTAPTPFLTKTFNLVEDPSTNDVISWNEDGSSFVVWNQTDFAKDLLPKHFKHNNFSSFVRQLNTYGFKKVVPDRWEFSNEFFRRGEKRLLRDIQRRKLTPQAAVSPPRPEVAAAQTAKTAVSPSSSGEVSPSSWTGSGGLSEELLEENERLRSENIQLSRELAQMKSLYGNIFGLMSNYAGASSSSHEMMMDIEEEASPRLFGVSIGLKRTRSEGVHVKTVSTAAEETPWLRHYNLANQRVCN

>BnaCnng56320D (BnaHsf45)

MTAAQRSVPAPFLSKTYQLVDDHSTDGVVSWNEDGTAFVVWETAEFAKDLLPKYFKHNNFSSFIRQLNTYGFRKTVPDKWEFANDHFRRGQEDLLSEIRRRKAVIAAAAEPNSAGDDDSMSSPGSKNPGSVENMVADLSGENEKLKRENDSLSSELAAAKRLRDELVTFLTDQLKVGPEQIDQMVKGGKFKPTVTEYCSSDEVSDCEGCGGDGEEKEGAGEGLKLFGVWVKGERKKRGRDEKNFVVGGSHRTTDIKNVDFHAPLWKSSKVCN

>AT4G17750 (AtHsfA1A)

MFVNFKYFSFFIRTKMDGVTGGGTNIGEAVTAPPPRNPHPATLLNANSLPPPFLSKTYDMVEDPATDAIVSWSPTNNSFIVWDPPEFSRDLLPKYFKHNNFSSFVRQLNTYGFRKVDPDRWEFANEGFLRGQKHLLKKISRRKSVQGHGSSSSNPQSQQLSQGQGSMAALSSCVEVGKFGLEEEVEQLKRDKNVLMQELVKLRQQQQTTDNKLQVLVKHLQVMEQRQQQIMSFLAKAVQNPTFLSQFIQKQTDSNMHVTEANKKRRLREDSTAATESNSHSHSLEASDGQIVKYQPLRNDSMMWNMMKTDDKYPFLDGFSSPNQVSGVTLQEVLPITSGQSQAYASVPSGQPLSYLPSTSTSLPDTIMPETSQIPQLTRESINDFPTENFMDTEKNVPEAFISPSPFLDGGSVPIQLEGIPEDPEIDELMSNFEFLEEYMPESPVFGDATTLENNNNNNNNNNNNNNNNNNNNTNGRHMDKLIEELGLLTSETEH

>AT5G16820 (AtHsfA1B)

MESVPESVPSPNSNTPSIPPPVNSVPPFLSKTYDMVDDPLTNEVVSWSSGNNSFVVWSAPEFSKVLLPKYFKHNNFSSFVRQLNTYGFRKVDPDRWEFANEGFLRGRKQLLKSIVRRKPSHVQQNQQQTQVQSSSVGACVEVGKFGIEEEVERLKRDKNVLMQELVRLRQQQQATENQLQNVGQKVQVMEQRQQQMMSFLAKAVQSPGFLNQLVQQNNNDGNRQIPGSNKKRRLPVDEQENRGDNVANGLNRQIVRYQPSINEAAQNMLRQFLNTSTSPRYESVSNNPDSFLLGDVPSSTSVDNGNPSSRVSGVTLAEFSPNTVQSATNQVPEASLAHHPQAGLVQPNIGQSPAQGAAPADSWSPEFDLVGCETDSGECFDPIMAVLDESEGDAISPEGEGKMNELLEGVPKLPGIQDPFWEQFFSVELPAIADTDDILSGSVENNDLVLEQEPNEWTRNEQQMKYLTEQMGLLSSEAQRK

>AT1G32330 (AtHsfA1D)

MDVSKVTTSDGGGDSMETKPSPQPQPAAILSSNAPPPFLSKTYDMVDDHNTDSIVSWSANNNSFIVWKPPEFARDLLPKNFKHNNFSSFVRQLNTYGFRKVDPDRWEFANEGFLRGQKHLLQSITRRKPAHGQGQGHQRSQHSNGQNSSVSACVEVGKFGLEEEVERLKRDKNVLMQELVRLRQQQQSTDNQLQTMVQRLQGMENRQQQLMSFLAKAVQSPHFLSQFLQQQNQQNESNRRISDTSKKRRFKRDGIVRNNDSATPDGQIVKYQPPMHEQAKAMFKQLMKMEPYKTGDDGFLLGNGTSTTEGTEMETSSNQVSGITLKEMPTASEIQSSSPIETTPENVSAASEATENCIPSPDDLTLPDFTHMLPENNSEKPPESFMEPNLGGSSPLLDPDLLIDDSLSFDIDDFPMDSDIDPVDYGLLERLLMSSPVPDNMDSTPVDNETEQEQNGWDKTKHMDNLTQQMGLLSPETLDLSRQNP

>AT3G02990 (AtHsfA1E)

EMGTVCESVATAKSSTAVMSSIPPFLSKTYDMVDDPLTDDVVSWSSGNNSFVVWNVPEFAKQFLPKYFKHNNFSSFVRQLNTYGFRKVDPDRWEFANEGFLRGQKQILKSIVRRKPAQVQPPQQPQVQHSSVGACVEVGKFGLEEEVERLQRDKNVLMQELVRLRQQQQVTEHHLQNVGQKVHVMEQRQQQMMSFLAKAVQSPGFLNQFSQQSNEANQHISESNKKRRLPVEDQMNSGSHGVNGLSRQIVRYQSSMNDATNTMLQQIQQMSNAPSHESLSSNNGSFLLGDVPNSNISDNGSSSNGSPEVTLADVSSIPAGFYPAMKYHEPCETNQVMETNLPFSQGDLLPPTQGAAASGSSSSDLVGCETDNGECLDPIMAVLDGALELEADTLNELLPEVQDSFWEQFIGESPVIGETDELISGSVENELILEQLELQSTLSNVWSKNQQMNHLTEQMGLLTSDALRK

>AT2G26150 (AtHsfA2)

MEELKVEMEEETVTFTGSVAASSSVGSSSSPRPMEGLNETGPPPFLTKTYEMVEDPATDTVVSWSNGRNSFVVWDSHKFSTTLLPRYFKHSNFSSFIRQLNTYGFRKIDPDRWEFANEGFLAGQKHLLKNIKRRRNMGLQNVNQQGSGMSCVEVGQYGFDGEVERLKRDHGVLVAEVVRLRQQQHSSKSQVAAMEQRLLVTEKRQQQMMTFLAKALNNPNFVQQFAVMSKEKKSLFGLDVGRKRRLTSTPSLGTMEENLLHDQEFDRMKDDMEMLFAAAIDDEANNSMPTKEEQCLEAMNVMMRDGNLEAALDVKVEDLVGSPLDWDSQDLHDMVDQMGFLGSEP

>AT5G03720 (AtHsfA3)

MSPKKDAVSKPTPISVPVSRRSDIPGSLYVDTDMGFSGSPLPMPLDILQGNPIPPFLSKTFDLVDDPTLDPVISWGLTGASFVVWDPLEFARIILPRNFKHNNFSSFVRQLNTYGFRKIDTDKWEFANEAFLRGKKHLLKNIHRRRSPQSNQTCCSSTSQSQGSPTEVGGEIEKLRKERRALMEEMVELQQQSRGTARHVDTVNQRLKAAEQRQKQLLSFLAKLFQNRGFLERLKNFKGKEKGGALGLEKARKKFIKHHQQPQDSPTGGEVVKYEADDWERLLMYDEETENTKGLGGMTSSDPKGKNLMYPSEEEMSKPDYLMSFPSPEGLIKQEETTWSMGFDTTIPSFSNTDAWGNTMDYNDVSEFGFAAETTSDGLPDVCWEQFAAGITETGFNWPTGDDDDNTPMNDP

>AT4G18880 (AtHsfA4A)

MDENNHGVSSSSLPPFLTKTYEMVDDSSSDSIVSWSQSNKSFIVWNPPEFSRDLLPRFFKHNNFSSFIRQLNTYGFRKADPEQWEFANDDFVRGQPHLMKNIHRRKPVHSHSLPNLQAQLNPLTDSERVRMNNQIERLTKEKEGLLEELHKQDEEREVFEMQVKELKERLQHMEKRQKTMVSFVSQVLEKPGLALNLSPCVPETNERKRRFPRIEFFPDEPMLEENKTCVVVREEGSTSPSSHTREHQVEQLESSIAIWENLVSDSCESMLQSRSMMTLDVDESSTFPESPPLSCIQLSVDSRLKSPPSPRIIDMNCEPDGSKEQNTVAAPPPPPVAGANDGFWQQFFSENPGSTEQREVQLERKDDKDKAGVRTEKCWWNSRNVNAITEQLGHLTSSERS

>AT5G45710 (AtHsfA4C)

MDENNGGSSSLPPFLTKTYEMVDDSSSDSVVAWSENNKSFIVKNPAEFSRDLLPRFFKHKNFSSFIRQLNTYGFRKVDPEKWEFLNDDFVRGRPYLMKNIHRRKPVHSHSLVNLQAQNPLTESERRSMEDQIERLKNEKEGLLAELQNQEQERKEFELQVTTLKDRLQHMEQHQKSIVAYVSQVLGKPGLSLNLENHERRKRRFQENSLPPSSSHIEQVEKLESSLTFWENLVSESCEKSGLQSSSMDHDAAESSLSIGDTRPKSSKIDMNSEPPVTVTAPAPKTGVNDDFWEQCLTENPGSTEQQEVQSERRDVGNDNNGNKIGNQRTYWWNSGNVNNITEKAS

>AT4G13980 (AtHsfA5)

MNGALGNSSASVSGGEGAGGPAPFLVKTYEMVDDSSTDQIVSWSANNNSFIVWNHAEFSRLLLPTYFKHNNFSSFIRQLNTYGFRKIDPERWEFLNDDFIKDQKHLLKNIHRRKPIHSHSHPPASSTDQERAVLQEQMDKLSREKAAIEAKLLKFKQQKVVAKHQFEEMTEHVDDMENRQKKLLNFLETAIRNPTFVKNFGKKVEQLDISAYNKKRRLPEVEQSKPPSEDSHLDNSSGSSRRESGNIFHQNFSNKLRLELSPADSDMNMVSHSIQSSNEEGASPKGILSGGDPNTTLTKREGLPFAPEALELADTGTCPRRLLLNDNTRVETLQQRLTSSEETDGSFSCHLNLTLASAPLPDKTASQIAKTTLKSQELNFNSIETSASEKNRGRQEIAVGGSQANAAPPARVNDVFWEQFLTERPGSSDNEEASSTYRGNPYEEQEEKRNGSMMLRNTKNIEQLTL

>AT5G43840 (AtHsfA6A)

MDYNLPIPLEGLKETPPTAFLTKTYNIVEDSSTNNIVSWSRDNNSFIVWEPETFALICLPRCFKHNNFSSFVRQLNTYGFKKIDTERWEFANEHFLKGERHLLKNIKRRKTSSQTQTQSLEGEIHELRRDRMALEVELVRLRRKQESVKTYLHLMEEKLKVTEVKQEMMMNFLLKKIKKPSFLQSLRKRNLQGIKNREQKQEVISSHGVEDNGKFVKAEPEEYGDDIDDQCGGVFDYGDELHIASMEHQGQGEDEIEMDSEGIWKGFVLSEEEMCDLVEHFI

>AT3G22830 (AtHsfA6B)

MDPSFRFIKEEFPAGFSDSPSPPSSSSYLYSSSMAEAAINDPTTLSYPQPLEGLHESGPPPFLTKTYDLVEDSRTNHVVSWSKSNNSFIVWDPQAFSVTLLPRFFKHNNFSSFVRQLNTYGFRKVNPDRWEFANEGFLRGQKHLLKNIRRRKTSNNSNQMQQPQSSEQQSLDNFCIEVGRYGLDGEMDSLRRDKQVLMMELVRLRQQQQSTKMYLTLIEEKLKKTESKQKQMMSFLARAMQNPDFIQQLVEQKEKRKEIEEAISKKRQRPIDQGKRNVEDYGDESGYGNDVAASSSALIGMSQEYTYGNMSEFEMSELDKLAMHIQGLGDNSSAREEVLNVEKGNDEEEVEDQQQGYHKENNEIYGEGFWEDLLNEGQNFDFEGDQENVDVLIQQLGYLGSSSHTN

>AT3G51910 (AtHsfA7A)

MMNPFLPEGCDPPPPPQPMEGLHENAPPPFLTKTFEMVDDPNTDHIVSWNRGGTSFVVWDLHSFSTILLPRHFKHSNFSSFIRQLNTYGFRKIEAERWEFANEEFLLGQRQLLKNIKRRNPFTPSSSPSHDACNELRREKQVLMMEIVSLRQQQQTTKSYIKAMEQRIEGTERKQRQMMSFLARAMQSPSFLHQLLKQRDKKIKELEDNESAKRKRGSSSMSELEVLALEMQGHGKQRNMLEEEDHQLVVERELDDGFWEELLSDESLASTS

>AT3G63350 (AtHsfA7B)

MDPSSSSRARSMPPPVPMEGLQEAGPSPFLTKTFEMVGDPNTNHIVSWNRGGISFVVWDPHSFSATILPLYFKHNNFSSFVRQLNTYGFRKIEAERWEFMNEGFLMGQRDLLKSIKRRTSSSSPPSLNYSQSQPEAHDPGVELPQLREERHVLMMEISTLRQEEQRARGYVQAMEQRINGAEKKQRHMMSFLRRAVENPSLLQQIFEQKRDREEAAMIDQAGLIKMEEVEHLSELEALALEMQGYGRQRTDGVERELDDGFWEELLMNNENSDEEEANVKQD

>AT1G67970 (AtHsfA8)

MVKSTDGGGGSSSSSSVAPFLRKCYDMVDDSTTDSIISWSPSADNSFVILDTTVFSVQLLPKYFKHSNFSSFIRQLNIYGFRKVDADRWEFANDGFVRGQKDLLKNVIRRKNVQSSEQSKHESTSTTYAQEKSGLWKEVDILKGDKQVLAQELIKVRQYQEVTDTKMLHLEDRVQGMEESQQEMLSFLVMVMKNPSLLVQLLQPKEKNTWRKAGEGAKIVEEVTDEGESNSYGLPLVTYQPPSDNNGTAKSNSNDVNDFLRNADMLKFCLDENHVPLIIPDLYDDGAWEKLLLLSPSRKKTKKQENIVKKGKDDLTLEEEEEDGTMELDKSYMLKLISEEMEKPDDFEFGQLTPERSRNLEILTEQMELLASNE

>AT5G54070 (AtHsfA9)

MTAIPNVVDIESSSSSLCQETATETVTVERGSSDSSSKPDDVVLLIKEEEDDAVNLSLGFWKLHEIGLITPFLRKTFEIVDDKVTDPVVSWSPTRKSFIIWDSYEFSENLLPKYFKHKNFSSFIRQLNSYGFKKVDSDRWEFANEGFQGGKKHLLKNIKRRSKNTKCCNKEASTTTTETEVESLKEEQSPMRLEMLKLKQQQEESQHQMVTVQEKIHGVDTEQQHMLSFFAKLAKDQRFVERLVKKRKMKIQRELEAAEFVKKLKLLQDQETQKNLLDVEREFMAMAATEHNPEPDILVNNQSGNTRCQLNSEDLLVDGGSMDVNGRIEIE

>AT4G36990 (AtHsfB1)

MTAVTAAQRSVPAPFLSKTYQLVDDHSTDDVVSWNEEGTAFVVWKTAEFAKDLLPQYFKHNNFSSFIRQLNTYGFRKTVPDKWEFANDYFRRGGEDLLTDIRRRKSVIASTAGKCVVVGSPSESNSGGGDDHGSSSTSSPGSSKNPGSVENMVADLSGENEKLKRENNNLSSELAAAKKQRDELVTFLTGHLKVRPEQIDKMIKGGKFKPVESDEESECEGCDGGGGAEEGVGEGLKLFGVWLKGERKKRDRDEKNYVVSGSRMTEIKNVDFHAPLWKSSKVCN

>AT5G62020 (AtHsfB2A)

MNSPPVDAMITGESSSQRSIPTPFLTKTFNLVEDSSIDDVISWNEDGSSFIVWNPTDFAKDLLPKHFKHNNFSSFVRQLNTYGFKKVVPDRWEFSNDFFKRGEKRLLREIQRRKITTTHQTVVAPSSEQRNQTMVVSPSNSGEDNNNNQVMSSSPSSWYCHQTKTTGNGGLSVELLEENEKLRSQNIQLNRELTQMKSICDNIYSLMSNYVGSQPTDRSYSPGGSSSQPMEFLPAKRFSEMEIEEEEEASPRLFGVPIGLKRTRSEGVQVKTTAVVGENSDEETPWLRHYNRTNQRVCN

>AT4G11660 (AtHsfB2B)

MPGEQTGETPTVAGVGGGGAGCSAGNSGGSSGCGAGGGGGGSGGGGGGGGDSQRSIPTPFLTKTYQLVEDPVYDELISWNEDGTTFIVWRPAEFARDLLPKYFKHNNFSSFVRQLNTYGFRKVVPDRWEFSNDCFKRGEKILLRDIQRRKISQPAMAAAAAAAAAAVAASAVTVAAVPVVAHIVSPSNSGEEQVISSNSSPAAAAAAIGGVVGGGSLQRTTSCTTAPELVEENERLRKDNERLRKEMTKLKGLYANIYTLMANFTPGQEDCAHLLPEGKPLDLLPERQEMSEAIMASEIETGIGLKLGEDLTPRLFGVSIGVKRARREEELGAAEEEDDDRREAAAQEGEQSSDVKAEPMEENNSGNHNGSWLELGK

>AT2G41690 (AtHsfB3)

MEDAGEHLRCNDNVNDEERLPLEFMIGNSTSTAELQPPPPFLVKTYKVVEDPTTDGVISWNEYGTGFVVWQPAEFARDLLPTLFKHCNFSSFVRQLNTYGFRKVTTIRWEFSNEMFRKGQRELMSNIRRRKSQHWSHNKSNHQVVPTTTMVNQEGHQRIGIDHHHEDQQSSATSSSFVYTALLDENKCLKNENELLSCELGKTKKKCKQLMELVERYRGEDEDATDESDDEEDEGLKLFGVKLE

>AT1G46264 (AtHsfB4)

MAMMVENSYGGYGGGGGERIQLMVEGQGKAVPAPFLTKTYQLVDDPATDHVVSWGDDDTTFVVWRPPEFARDLLPNYFKHNNFSSFVRQLNTYGFRKIVPDRWEFANEFFKRGEKHLLCEIHRRKTSQMIPQQHSPFMSHHHAPPQIPFSGGSFFPLPPPRVTTPEEDHYWCDDSPPSRPRVIPQQIDTAAQVTALSEDNERLRRSNTVLMSELAHMKKLYNDIIYFVQNHVKPVAPSNNSSYLSSFLQKQQQQQPPTLDYYNTATVNATNLNALNSSPPTSQSSITVLEDDHTNHHDQSNMRKTKLFGVSLPSSKKRSHHFSDQTSKTRLVLDQSDLALNLMTASTR

>AT3G24520 (AtHsfC)

MEDDNSNNNNNNNVIAPFIVKTYQMVNDPSTDWLITWGPAHNSFIVVDPLDFSQRILPAYFKHNNFSSFVRQLNTYGFRKVDPDRWEFANEHFLRGQKHLLNNIARRKHARGMYGQDLEDGEIVREIERLKEEQRELEAEIQRMNRRIEATEKRPEQMMAFLYKVVEDPDLLPRMMLEKERTKQQQQVSDKKKRRVTMSTVKSEEEEVEEDEGRVFRVMSSSTPSPSSTENLYRNHSPDGWIVPMTQGQFGSYETGLVAKSMLSNSTSSTSSSLTSTFSLPESVNGGGGGGCGSIQGERRYKETATFGGVVESNPPTTPPYPFSLFRGGF
