# Supplementary figures and images for: Systematic Analysis of Hsf Family Genes in the Brassica napus Genome Reveals Novel Responses to Heat, Drought and High CO2 Stresses
Source: Front Plant Sci. 2017 Jul 6;8:1174. doi: 10.3389/fpls.2017.01174 (PMC5498556; doi:10.3389/fpls.2017.01174)

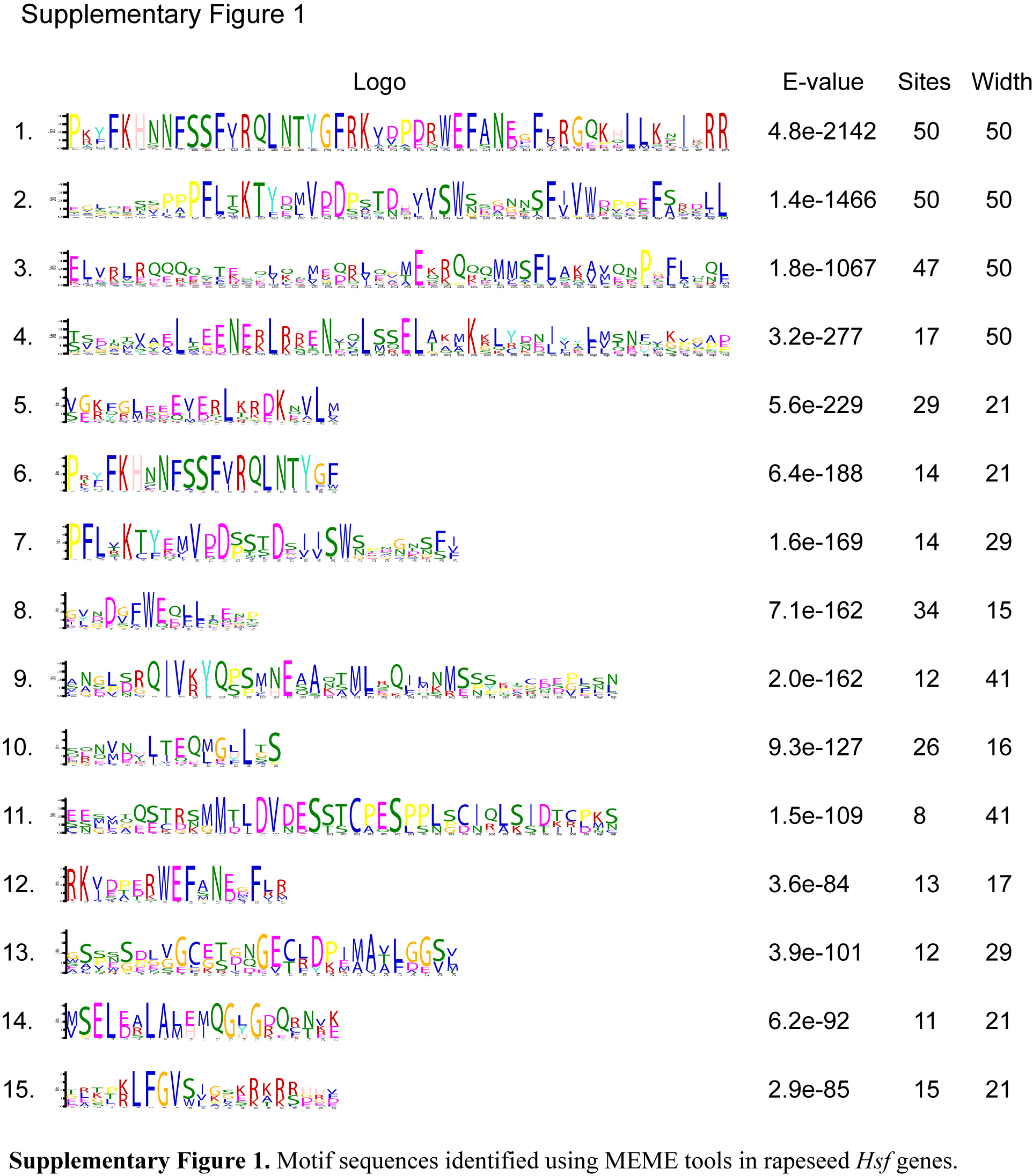

Supplement: Supplementary file 7 [file Image_1.JPEG]
